# Hypothesis-driven dragging of transcriptomic data to analyze proven targeted pathways in *Rhinella arenarum* larvae exposed to organophosphorus pesticides

**DOI:** 10.1038/s41598-022-21748-6

**Published:** 2022-10-21

**Authors:** Natalia S. Pires, Cecilia I. Lascano, Julia Ousset, Danilo G. Ceschin, Andrés Venturino

**Affiliations:** 1 Centro de Investigaciones en Toxicología Ambiental y Agrobiotecnología del Comahue (CITAAC), Universidad Nacional del Comahue-CONICET, 8300 Buenos Aires 1400, Neuquén Argentina; 2 Centro de Investigación en Medicina Traslacional “Severo R. Amuchástegui” (CIMETSA), Vinculado al Instituto de Investigación Médica Mercedes y Martín Ferreyra (CONICET-UNC), Instituto Universitario de Ciencias Biomédicas de Córdoba (IUCBC), Av. Naciones Unidas 420, X5016KEJ Córdoba, Argentina

**Keywords:** Biochemistry, Computational biology and bioinformatics, Data mining, Molecular biology, Transcriptomics

## Abstract

Transcriptional analysis of the network of transcription regulators and target pathways in exposed organisms may be a hard task when their genome remains unknown. The development of hundreds of qPCR assays, including primer design and normalization of the results with the appropriate housekeeping genes, seems an unreachable task. Alternatively, we took advantage of a whole transcriptome study on *Rhinella arenarum* larvae exposed to the organophosphorus pesticides azinphos-methyl and chlorpyrifos to evaluate the transcriptional effects on a priori selected groups of genes. This approach allowed us to evaluate the effects on hypothesis-selected pathways such as target esterases, detoxifying enzymes, polyamine metabolism and signaling, and regulatory pathways modulating them. We could then compare the responses at the transcriptional level with previously described effects at the enzymatic or metabolic levels to obtain global insight into toxicity–response mechanisms. The effects of both pesticides on the transcript levels of these pathways could be considered moderate, while chlorpyrifos-induced responses were more potent and earlier than those elicited by azinphos-methyl. Finally, we inferred a prevailing downregulation effect of pesticides on signaling pathways and transcription factor transcripts encoding products that modulate/control the polyamine and antioxidant response pathways. We also tested and selected potential housekeeping genes based on those reported for other species. These results allow us to conduct future confirmatory studies on pesticide modulation of gene expression in toad larvae.

## Introduction

The Upper Valley of Rio Negro and Neuquén (North Patagonia) is a region with an intensive fruit production. Because of the massive application of pesticides used for pest control, many aquatic vertebrates and invertebrates living in backwaters and irrigation channels are challenged. Organophosphorus (OP) insecticides have been the pest controllers most frequently and heavily applied in fruit orchards of the region, being azinphos-methyl (AZM) and chlorpyrifos (CPF) the main products successively used over the last two decades^1,2^. Both OP pesticides have been found in superficial and groundwaters from this region with a high frequency (80% or more) and environmental concentrations reaching 0.02–0.08 mg/L of pesticide^2–4^. Usually, the reproductive season of amphibians coincides with the period of pesticide application during Springtime. This is the case for the autochthonous amphibian *Rhinella arenarum*, whose embryos and larvae are potentially exposed to transient but high concentrations of OP, reaching the order of 1 mg/L^2,5^, as well as for other aquatic vertebrates^6–11^. Malformations can occur at sublethal concentrations, usually accompanied by swimming alterations and other impairments in movement. Besides the main targets of OP, i.e., synaptic and motor-plate acetylcholinesterases, other targets are involved in sublethal and long-term effects caused by OP. The effects of OP on these secondary biochemical and molecular targets have been described in *R. arenarum* and other amphibians^12,13^. Briefly, AZM and CPF affect the antioxidant defenses, reducing glutathione content, and enzyme activities of such as catalase, glutathione reductase and glutathione peroxidase while superoxide dismutase activity is either reduced or increased depending on toxicant levels^7,8,11,14–17^. Organophosphorus pesticides also affect the detoxifying systems metabolizing them, inhibiting carboxylesterase activity, a known buffering system protecting the main target acetylcholinesterase, while increasing glutathione-S-transferase activity in general, as well as mixed-function oxidases activity^8,11,16–18^. Polyamine metabolism, related to embryonic development, is also differentially affected by OP depending on pesticide concentrations, exposure duration and the chemical structure *per se*, altering polyamine levels, their synthesis rate through the key enzyme ornithine decarboxylase, and through degrading oxidase enzymes^7,10,19,20^. Finally, several key regulatory pathways involved in amphibian development, detoxifying and oxidative stress responses are differentially affected by OP at the protein content and/or phosphorylation levels depending on exposure conditions, including Protein Kinase C, Mitogen-Activated-Protein Kinase pathway (Mek, Erk), cFos and cJun^7,21^. These biochemical and molecular targets underlie, at least in part, the morphological and behavioral effects of chemicals on amphibians, leading to their particular susceptibility to contaminants during the embryonic development and metamorphosis as one of the probable causes of their decline in the natural habitats^13,22^.

When evaluating the risks of exposure to a contaminant for any species, it is desirable to find early-response biomarkers capable of anticipating irreversible damage that may occur at later stages of development^23^. Molecular targets, effectors, or modulators of toxicant effects are among early-response biomarkers. However, their assessment in autochthonous, nonmodel species such as *R. arenarum* is difficult due to the lack of sequenced genomes that allow primer design and transcript analysis, as well as the specific antibodies necessary to support protein expression analysis^12^. In these cases, toxicogenomic and particularly transcriptomic analyses through bulk RNA sequencing (RNA-Seq) are powerful tools that enable screening for effects and responses to a toxicant and therefore allow further molecular studies^24,25^. RNA-Seq is a highly sensitive and accurate tool for measuring expression across the transcriptome, allowing researchers to detect changes that otherwise would go unnoticed. It is possible to quantify abundance levels or relative changes for each transcript during a specific developmental stage or under a specific treatment^26^. In the case of nonmodel organisms such as *R. arenarum*, RNA-Seq technology allows the obtention of genomic information through a relatively accessible process from an economic point of view and considering the cost-benefit relationship^25^.

We have been studying the effects of OP pesticides in the development of the common toad *R. arenarum*, focusing on target and detoxifying enzymes, polyamine pathway and signaling, at protein, activity and metabolite levels. However, our advances at the transcript expression level were slow and challenging until we were able to develop a transcriptome study. In the context of this RNA-Seq and transcriptomic analysis performed on *R. arenarum* larvae exposed to AZM and CPF^25^, we were able to perform hypothesis-driven data analysis processing on *a priori* selected mRNA transcripts. This work was done in parallel with the whole transcriptome bioinformatic (big data) analysis, whose results are to be published elsewhere. The aims of the study were a) to compare transcript expression levels in genes from pathways that have been previously recognized as impacted by OP pesticides and that have also been studied at the biochemical, metabolite or physiological level in *R. arenarum* to obtain a more comprehensive mechanism of toxicity and response based on the involved regulatory pathways and their crosstalk, and b) to test a set of potentially adequate housekeeping genes in *R. arenarum* larval stages for future quantitative PCR studies.

## Results

### Gene selection from annotated transcripts for OP effect analysis.

**Figure 1:**
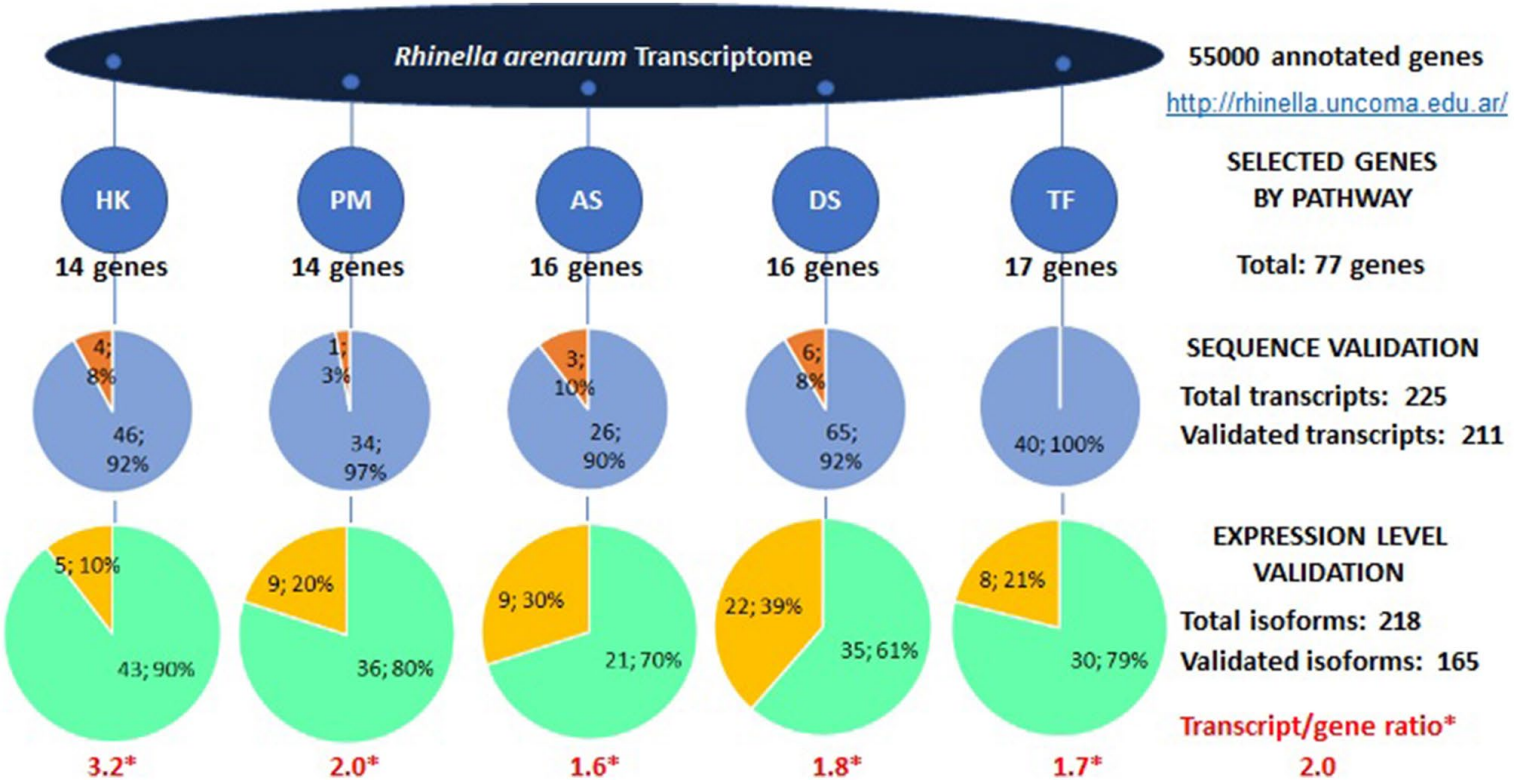
Gene selection process by metabolic pathway. Our starting point was the database with 55,000 annotated genes from the *R.* *arenarum* transcriptome previously assembled by us^13^. The first step was a hypothesis-driven selection of annotated genes from five pathways recognized as affected by organophosphorus pesticides at enzyme activity or metabolic product levels: *PM* polyamine metabolism, *AS* antioxidant system, *DS* detoxifying systems, *TF* transcription factors, signaling pathways; and Housekeeping (HK) genes tested in amphibians. The next step was checking the selected transcript sequences and their predicted amino acid sequence identities with the annotated genes by BLASTN and BLASTP, from which about 94% of isoforms were confirmed (light blue vs. brown slices). The last step was checking the transcription levels for each isoform to discard erratic low values, using the differential expression data corrected by the trimmed mean of M-values (TMM) normalization method, accepting 76% of them (light green vs. yellow slices). Note that, on average, there were 2 accepted transcripts per annotated gene.

We used a list of the available gene transcripts in *R. arenarum* published by us in the database from massive RNA transcript sequencing and gene annotation (http://rhinella.uncoma.edu.ar/;^25^). We selected a group of 14 potential housekeeping genes, considering those currently proposed for mRNA expression normalization in *Xenopus laevis*^27–29^ (Table S1, Group A). A considerable number of annotated genes could be included in the proposed pathways, which were selected on the basis of available information about OP effects at the biochemical level in amphibians^7,8,10^ and other vertebrates: polyamine metabolism genes, (Group B, 14 genes); antioxidant response genes (Group C, 15 genes); OP- metabolizing, primary- and secondary-target enzyme genes (Group D, 16 genes); and gene expression regulators, transcription factors and phosphorylation cascade effectors corresponding to the Mitogen-Activated protein kinase pathway, Transcription factor AP-1, Aryl hydrocarbon receptor (AHR) pathway and Nuclear factor erythroid 2-related factor 2 antioxidant response pathway (Group E, 17 genes) (Fig. 1). The complete list of genes is detailed in Supplementary Table S1.

### Corroboration of gene annotation for selected transcripts

Alignments were carried out using the tools available in BLAST (BLASTN, BLASTX and BLASTP) to verify that the sequences of the selected transcripts effectively corresponded to the annotated genes. Sequences that produced a match greater than 50% in the vertebrate databases were selected for further analysis. A summary of this sequence validation is shown in Fig. 1, and the complete analysis is available in Supplementary Data File I. From a total of 225 transcript sequences that were compared to the corresponding annotated genes, 93.8% could be effectively confirmed; the best correspondence was obtained in group E covering transcription factors and signaling pathways with 100%, and the lowest percentage was approximately 90% for the group of antioxidant response transcripts (Group C).

### Validation of adequate transcription levels for filtered transcripts

Previously validated transcripts were analyzed to determine whether the transcription levels of treatment replicates were appropriate for statistical analysis. These levels resulted from the massive RNAseq amplification and quantitation of each transcript fragment in the different treatments. Fragments with zero expression levels in any sample and/or replicates with very low and erratic values, were considered as ‘not appropriate’ levels. A summary of the transcripts accepted in each group of selected genes is shown in Fig. 1. The raw data corresponding to all transcript fragments are available in Supplementary Data File II.

### Housekeeping gene stability

We carried out a transcriptional stability analysis of the selected potential housekeeping (HK) genes, comparing their variability within and between treatments. The respective TMM means, minimum and maximum expression values, standard deviations, median expressions, and percentual coefficients of variation (CV%) are presented in Table 1. We assumed a maximum CV% of 20% in the TMM as an acceptable limit for a transcript to be considered housekeeping. In this way, we were able to select 9 out of 12 transcripts as appropriate reference genes in the first larval stage of *R. arenarum*. The genes with the least and acceptable variations in group A were: *ef1a0*, *ef1ga*, *ef1d*, *ef1b*, *tuba*, *tubb*, *tubb4b*, *actb* and *rl8*.

**Table 1:** Comparison of distribution statistics and expression variation of transcripts proposed as HK in *R. arenarum* stage 25 larvae.

Gene ID	Gene name	Mean	Minimum	Maximum	SD	Median	CV %
*ef1a0*	Elongation factor 1-alpha, somatic form	7329.9	6610.7	8457.7	586.4	7265.4	8.00
*ef1b*	Elongation factor 1-beta	583.4	498.6	676.9	64.3	589.4	11.03
*ef1d*	Elongation factor 1-delta	922.1	800.7	1074.7	98.3	910.4	10.66
*ef1g-a*	Elongation factor 1-gamma-A	2993.7	2611.2	3344.1	252.9	2968.2	8.45
*g3pdh*	Glyceraldehyde-3-phosphate dehydrogenase	1822.0	1289.5	2425.1	411.3	1768.3	22.58
*rl8*	60S ribosomal protein L8	1108.0	883.6	1309.3	134.8	1131.6	12.16
*tuba*	Tubulin alpha chain	1829.9	1589.5	2014.7	142.9	1826.3	7.81
*tuba1*	Tubulin alpha-1 chain	146.1	98.1	195.5	31.8	142.1	21.74
*tubb*	Tubulin beta chain	518.4	350.2	586.8	70.4	526.8	13.58
*tubb4b*	Tubulin beta-4B chain	219.0	137.1	253.7	33.3	224.5	15.19
*actb*	Actin, cytoplasmic 1	363.6	301.7	394.4	37.2	380.3	10.22
*acta4*	Actin, alpha sarcomeric/skeletal	90.1	15.1	259.4	73.3	61.9	81.27

Data calculated from massive RNA-seq and transcriptomic analysis are expressed as TMM values. A limit of 20% for the CV is proposed to consider a transcript as an HK gene in expression analysis.

The transcripts belonging to tubulin *tuba1* and glyceraldehyde 3P dehydrogenase (*g3pdh*) showed a variability of approximately 22% in their expression levels, roughly in the exclusion limits we considered, and might be considered HK genes if a refined and specific analysis proves better results. In turn, sarcomeric actin *acta4* showed a 83% variation in expression, clearly indicating that it cannot be considered a HK gene in *R. arenarum* larvae for OP pesticide studies. The TMM values of the selected HK genes, rated with respect to their control values, were averaged for each treatment and used to standardize the TMM values of the transcripts corresponding to the genes of groups B, C, D and E (raw and standardized data shown in Supplementary Data File II).

### Effects of OP pesticides on the transcription of polyamine metabolism genes

**Figure 2:**
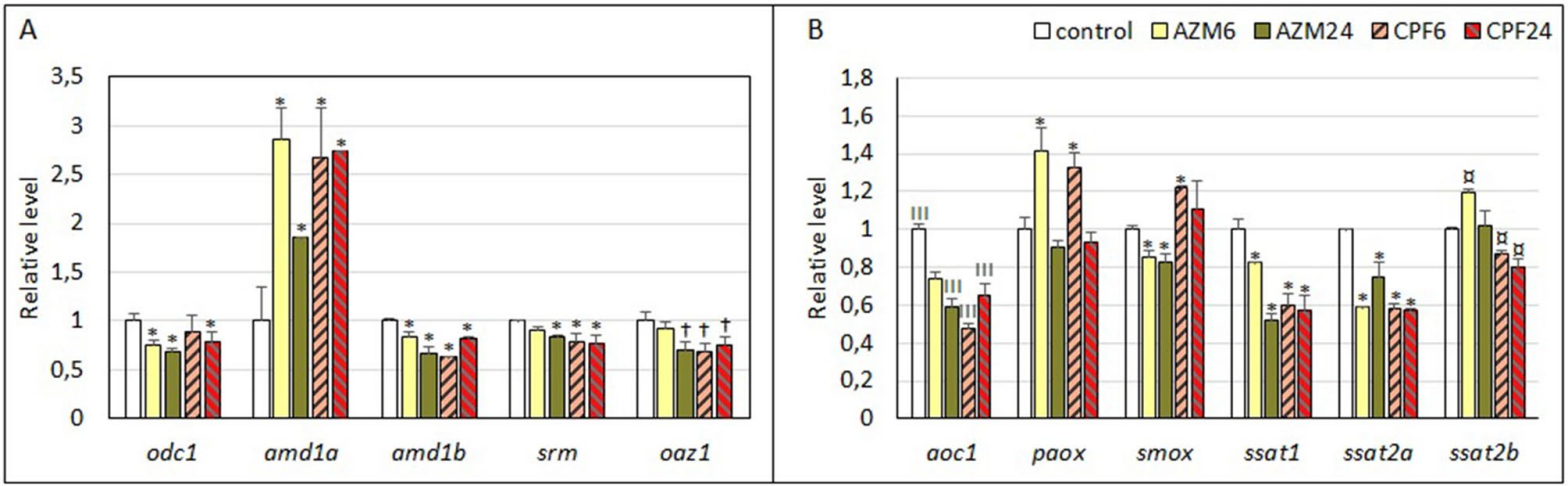
Expression levels of transcripts corresponding to polyamine metabolism genes in *R. arenarum* larvae. A, Genes related to polyamine synthesis and its regulation. B, genes related to polyamine degradation. Significance levels for Kruskal–Wallis and median tests, * *p* = 0.09; ¤ *p* = 0.08; †*p* = 0.04; III *p* = 0.0001. *AZM* azinphosmethyl, *CPF* chlorpyrifos, at 6 h and 24 h exposures; gene codes are detailed in the text.

OP pesticides mainly decreased the expression of genes related to polyamine synthesis (Fig. 2A). Although some differences could be observed between CPF and AZM and exposure times, decreases reaching 30–40% in expression levels were found in ornithine decarboxylase (*odc1*), S-adenosylmethionine decarboxylase proenzyme (*amd*, transcript *1-b*), spermidine synthase (*srm*), and ornithine decarboxylase antizyme (*oaz1*). The *amd* transcript *1-a* showed a significant increase of nearly 3 times the control values in larvae exposed to both OP pesticides. In turn, the ornithine decarboxylase regulator *oaz2* and the antizyme inhibitors *azin1* and *2* showed no significant or relevant changes (Supplementary Table S2).

In turn, polyamine-degrading enzymes showed more variable responses to OP pesticides (Fig. 2B). Expression of the *aoc1* transcript, corresponding to diamine oxidase enzyme, showed a significant inhibition of 25–40% by AZM treatment and 35–50% by CPF treatment. The *aoc2* transcript showed a similar trend with even deeper inhibition responses, but the difference was not significant (Table S2). The *aoc3* and *4* transcript isoforms showed variable and non-significant responses to OP pesticide exposure. Acetyl spermidine/spermine oxidase (*paox*) expression showed an induction trend of 40% with both OP pesticides at 6 h of exposure, while the expression of spermine acetylase transcripts (*sat1*, 2*-a*, 2*-b*) showed an inhibitory trend with CPF up to 40%. Spermine oxidase (*smox*) expression was barely inhibited (20%) by AZM and induced by CPF (20%, 6 h) (Figure 2B).

### Effects of OP pesticides on antioxidant response genes

**Figure 3:**
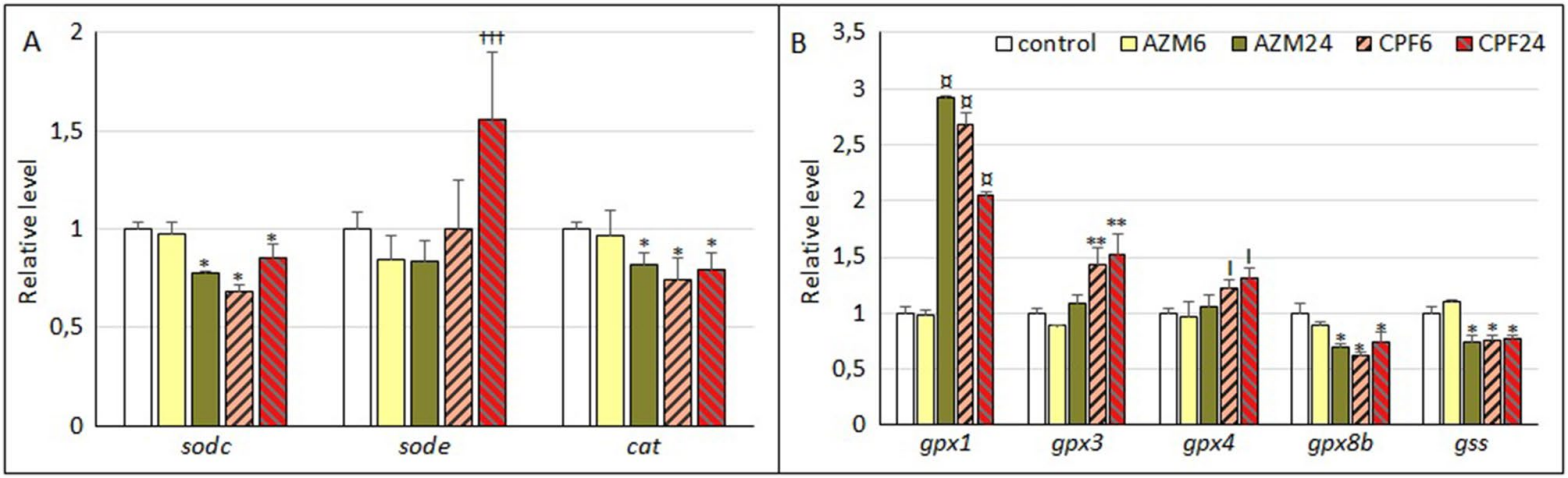
Expression levels of transcripts corresponding to antioxidant response genes in *R. arenarum* larvae. A, Superoxide dismutase (*sod*) and catalase (*cat*) genes. B, GSH-dependent antioxidant response and GSH synthesis genes. Significance levels for Kruskal–Wallis and median tests, *p = 0.09; ¤ *p* = 0.08; I *p* = 0.07; **p = 0.01; ***p = 0.003; †††*p* = 0.0007. *AZM* azinphosmethyl, *CPF* chlorpyrifos, at 6 h and 24 h exposures; gene codes are detailed in the text.

Superoxide dismutase transcripts displayed differential responses to OP pesticide treatments (Fig. 3A). The expression of *sodc* showed a decrease in response to AZM exposure at 24 h, and to CPF at 6 and 24 h of exposure (up to 30% inhibition) with respect to the control. On the other hand, *sode* expression was significantly induced by 60% in larvae exposed to CPF at 24 h. Catalase transcription (*cat*) was in turn slightly inhibited by AZM exposure at 24 h and by CPF up to 25%.

Glutathione-dependent peroxidase transcripts were mainly induced by OP pesticides: *gpx1* transcription was considerably induced by AZM at 24 h (3X) and by CPF at both times (up to 2.7X); *gpx3* transcription was induced to a lesser extent, but in a significant way, by CPF (1.5X), while *gpx4* also showed an induction of approximately 30% by CPF exposure. In turn, the transcript levels of *gpx8b* showed a tendency towards decrease in larvae exposed to AZM at 24 and CPF at 6 and 24 h, up to 60% of control expression values. Two other *gpx* transcripts, *gpx2* and *7*, as well as one GSH-reductase transcript (*gsr*), showed decreasing but not significant trends due to exposure to OP pesticides (Supplementary Table S2). Finally, a transcript for glutathione synthetase enzyme (*gss*) was downregulated by AZM exposure at 24 h and by CPF at both times, by approximately 25% (Fig. 3B).

### OP pesticide targets and detoxifying enzymes

**Figure 4:**
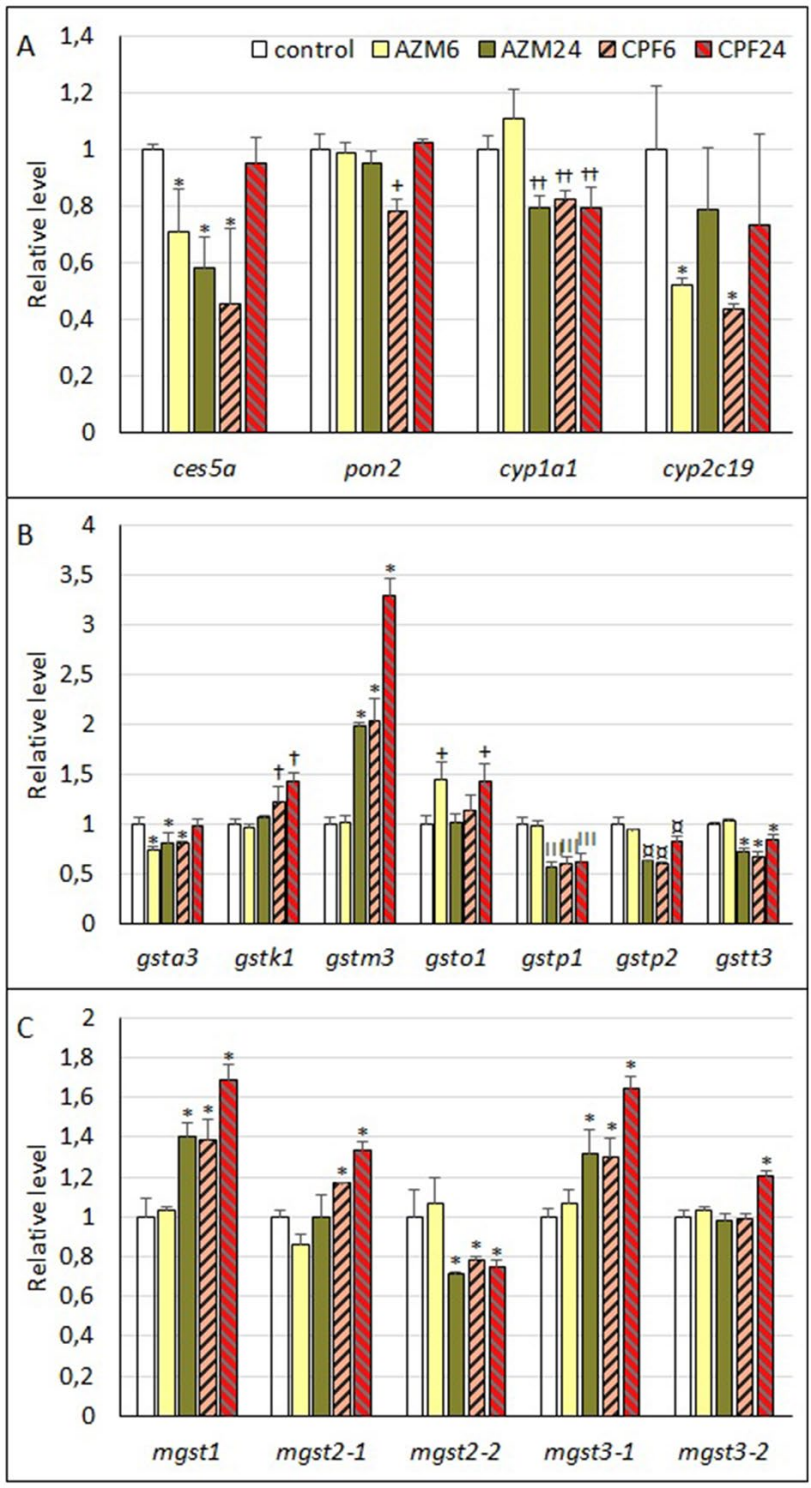
Expression levels of transcripts corresponding to OP-detoxifying enzyme genes in *R. arenarum* larvae. A, Esterases and Cytochrome P-450 isoforms. B, GSH-S Transferases (*gst*), cytosolic isoforms. C, GST, microsomal isoforms (*mgst*). Significance levels for Kruskal–Wallis and median tests, *p = 0.09; ¤ *p* = 0.08; + *p* = 0.05; † *p* = 0.04; †† *p* = 0.007; III *p* = 0.0001. *AZM* azinphosmethyl, *CPF* chlorpyrifos, at 6 h and 24 h exposures; gene codes are detailed in the text.

Although some fragments corresponding to cholinesterase transcripts (*bche*) were detected, their levels were very low and showed erratic responses (raw data in Supplementary Data File II). Among the esterase group, the carboxylesterase transcript *ces5a* showed a relevant decrease in larvae exposed to AZM at both 6 and 24 h, while in larvae exposed to CPF, a 50% decrease at 6 h was observed, returning to control values at 24 h (Fig. 4A). A similar pattern was observed for another carboxylesterase transcript, *ces3b*, but the effects were not significant (Supplementary Table S2). The serum paraoxonase/arylesterase-2 transcript *pon2* showed a small but significant decrease of approximately 20% in larvae exposed to CPF at 6 h with respect to the controls. For cytochrome P450 enzymes, only the transcripts related to OP metabolization were analyzed; *cyp1a1* transcript expression was significantly reduced by AZM at 24 h and by CPF exposure, although the effects were minor (20% inhibition). Cytochrome *cyp2c19* expression was more markedly inhibited by both OP pesticides after 6 h of treatment, reaching 50–60% inhibition compared to controls.

Glutathione-S-transferase (*gst*) transcripts were among the most abundant groups, as 15 different isoforms could be identified. The expressions of GST isoforms alpha (*gsta3*), pi (*gstp1* and *2*), and theta (*gstt3*) were downregulated by both OP pesticides, with transcription inhibitions ranging from 20 to 50% with respect to controls (Fig. 4B). Expression of the *gstp1* isoform showed the highest significant inhibition. Although the expression of GST Mu (*gstm1*) showed a decreasing trend, the effects were not significant (Table S2). On the other hand, the GST Kappa 1 transcript (*gstk1*) was significantly induced by CPF (45%). Also, *gstm3* showed the highest transcription induction values by CPF and by AZM at 24 h (2 X–3.3 X with respect to controls). GST omega 1 transcript (*gsto1*) also showed a significant increase of 45% in its expression for AZM at 6 h, and for CPF at 24 h.

Microsomal GST transcripts were also analyzed, finding increases in the expression levels of isoforms 1, 2 and 3 (*mgst1, mgst2*, *mgst3*, respectively, Fig. 4C). The *mgst1 *transcript was one of the most affected transcripts, with increases ranging from 40% (AZM-24 h; CPF-6 h) to 70% (CPF-24 h). One *mgst3* transcript (*mgst3-1*) showed roughly similar effects (30–60% increase), while a second transcript (*mgst3-2*) showed a moderate increase (20%) only for CPF at 24 h of exposure. We also identified two transcripts for *mgst2*, one of which showed a moderate increase after exposure to CPF (*mgst2-1*; up to 35%), and the other showed slight decreases of approximately 20% in its expression with respect to the controls (*mgst2-2*).

### OP effects on transcription factor- and signaling pathway-related genes

**Figure 5:**
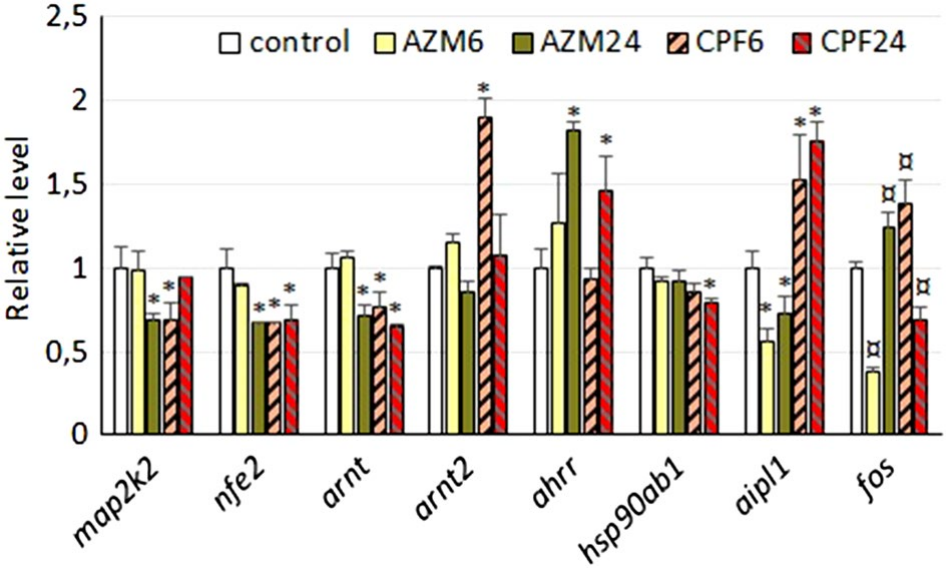
Expression levels of transcripts corresponding to transcription factors and signaling pathway genes in *R. arenarum* larvae. Significance levels for Kruskal–Wallis and median tests, *p = 0.09; ¤ *p* = 0.08. *AZM* azinphosmethyl, *CPF* chlorpyrifos, at 6 h and 24 h exposures; gene codes are detailed in the text.

The MAP kinase phosphorylation pathway appeared to be affected solely at the first level of regulation, since transcription of mitogen-activated protein kinase kinases (*map2k*) was downregulated; the *map2k2* transcript was decreased to 70% of the control values by CPF at 6 h and by AZM at 24 h (Fig. 5), while the *map2k1* transcript was only affected by CPF but not at a relevant level (Supplementary Table S2). Similarly, the mitogen-activated protein kinase p38 transcript (*mapk14)* was barely affected by both OP pesticides (approximately 12%), while the *junk* transcript (*mapk8)* remained unaffected. None of the detected JUN transcription factor transcripts (*jun1*, *junb*, *jund1*) were significantly affected by exposure to OP pesticides (Table S2). In turn, the transcription factor c-Fos, which associates with Jun as a heterodimer partner of activator protein-1 (AP1), showed a severe reduction in its transcription levels (*fos*) by AZM at 6 h of exposure (to 35% of control levels) but recovered at 24 h and surpassed control by 25%; CPF caused an inverse pattern, inducing *fos* transcription at 6 h (40%) but inhibiting it at 24 h (30%) (Fig. 5).

On the other hand, nuclear factor erythroid 2-related factor 2 (*nfe2*), linked to the antioxidant response through the antioxidant response element (ARE) pathway, showed a decrease in transcription of approximately 30% in larvae exposed to AZM for 24 h and in those exposed to CPF for 6 and 24 h compared to controls (Fig. 5).

Within the aryl hydrocarbon receptor (AhR) pathway, some differences were found for the nuclear translocator (*arnt*) and the aryl receptor repressor (*ahrr*). Two *arnt* transcripts were analyzed, the first showing a decrease of nearly 30% in larvae exposed to AZM at 24 h and to CPF at 6 and 24 h compared to controls. The second transcript, *arnt2*, showed a relevant induction in transcription of nearly 2X after 6 h of exposure to CPF. The *ahrr* transcript showed an increase in larvae after 24 h of exposure to AZM (80%) and CPF (40%) at 24 h with respect to controls. The *ahr* transcript itself showed a decreasing but not-significant pattern after exposure to CPF (Table S2). The transcript corresponding to the chaperone HSP90AB1 (*hsp90ab1)*, that binds AhR protein in the cytosol, showed a decrease of 20% after 24 h of exposure to CPF, while the transcript *aipl1*, corresponding to the cochaperone aryl-hydrocarbon-interacting protein-like-1, was downregulated by AZM (approximately 50%) but increased by CPF (up to 75%). Another isoform, *aip*, did not show effects, as well as the transcript for the third cochaperone prostaglandin E synthase-3, *ptges3* (Table S2).

### Overview of OP effects in hypothesis-selected pathway genes

**Figure 6:**
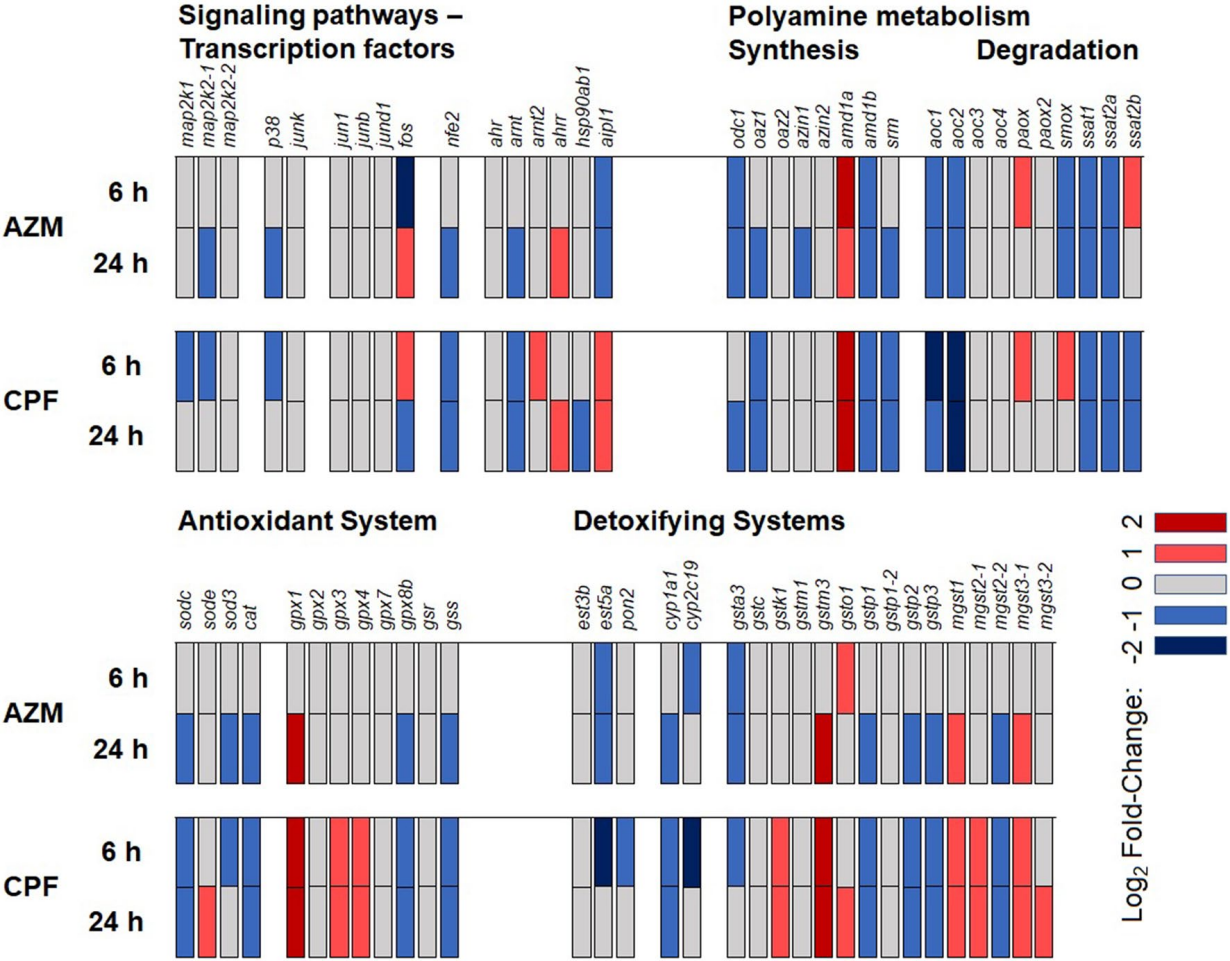
Heatmap representation of OP effects on selected gene expression in *R. arenarum* larvae. Data are presented for four groups of selected genes whose products or activities are known or suspected targets of OP pesticides. Larvae were exposed to azinphos-methyl (AZM) and chlorpyrifos (CPF) for 6 and 24 h. Gene codes are detailed in the text.

A comparison of the number of genes showing significant differential expression, their fold-changes, and their up or down regulation, was performed for the different OP treatments using a heatmap representation (Fig. 6). Exposure of *R. arenarum* larvae to AZM or CPF at sublethal concentrations and up to 24 h did not cause remarkable fold changes in gene expression in the selected pathways. Most of the selected genes did not show significant variations or changes were less than 2-fold; only eight transcripts showed changes between 2- and 4-fold. Exposure to AZM showed a weaker effect at 6 h compared to 24 h, as evidenced by the number of differentially affected transcript expression levels. In turn, the effects of CPF on *R. arenarum* were more potent, affecting most of the gene expression early at 6 h and sustaining the effects after 24 h of exposure. Another interesting feature was that transcriptional downregulation cases notably exceeded the upregulation ones, in a proportion of 3–1.

### Comparison /validation of statistical approaches and hypothesis-selected gene ranking in differential expression

We performed both edgeR pipeline statistical analysis on the whole transcriptome data, and nonparametric Kruskal–Wallis ANOVA and Median tests on the hypothesis-selected genes using the filtered database. The main reasons for using both approaches are that edgeR *a priori* considers all possible fragments for one annotated gene as different and that it is not feasible to filter erratic, usually low level-expressed transcripts; in turn, the specific filtering and checking of fragments enables their use as repeats in non-parametric tests. Keeping in mind that edgeR statistical analysis was performed on the raw, non-filtered database, after collapsing the different fragments for each annotated gene into one unique set (5 treatments in duplicate), we could compare the performances with nonparametric analysis in 42 of the 62 *a priori* selected genes. The coincidences between both statistical approaches extended to 66.7% of the analyzed transcripts, and when the treated- versus control pairs were considered, the result was similar, coinciding in 65.5% of all the cases. A detailed comparison of the transcripts can be found in Supplementary Table S3. From the 42 transcripts analyzed, 15 showed coincident statistical outputs in the four treatments, 7 transcripts did in 3 out of 4 cases, and 10 did in 2 of 4 cases; only one gene did not show coincidences at all. This fact highlights the need to carefully filter and validate transcripts, sequences, and expression levels when any selection is performed for a specific analysis. This may be particularly important in some instances, i.e., when 'top ten' differentially expressed genes (DEG) are considered.

**Figure 7:**
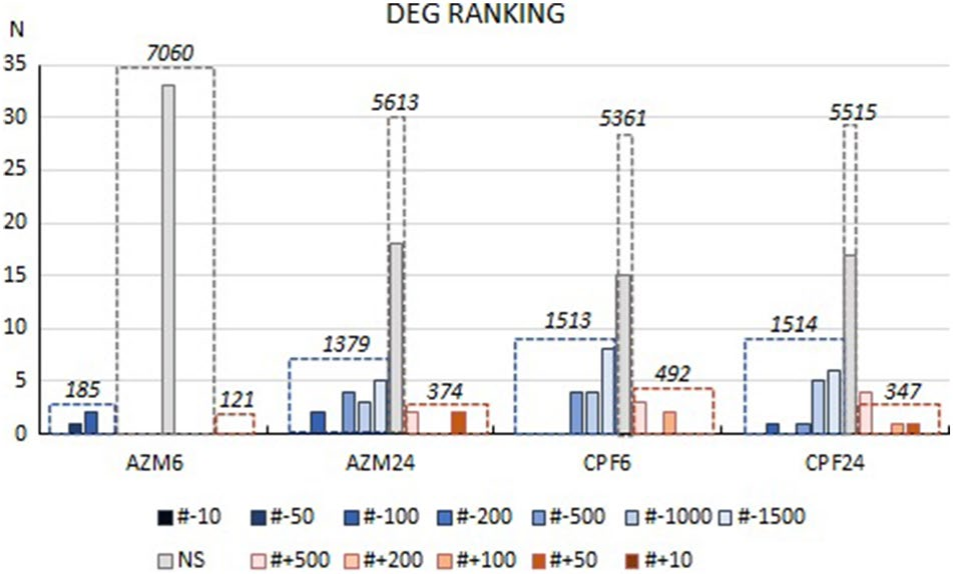
Ranking of differentially expressed genes (DEG) in *R. arenarum* larvae exposed to OP pesticides. The expression levels of annotated transcripts were normalized by TMM and analyzed by edgeR pipeline in treated-vs.-control pairs for azinphosmethyl (AZM) and chlorpyrifos (CPF) at 6 and 24 h-exposures. The significant DEG were further selected and ranked according to the log-fold changes. From the generated results, hypothesis-selected transcripts were identified, classified by their ranking into the different groups indicated in the figure bar codes either as downregulated (#-, in blue scale) or upregulated (# + , in red scale) DEG, or as NS according to the *p*-values, being N the counts in each category. Comparatively, the total downregulated, upregulated or NS/unaffected transcripts are shown by the dotted rectangles with the corresponding number of transcripts in italics. The data for hypothesis-selected transcripts is detailed in Table S3.

We next extracted the lists of significant DEG from edgeR- paired comparisons of pesticide-treated versus control transcript expressions and ranked them using the logarithm of their fold-changes. We determined the ranking positions for each hypothesis-selected gene showing significant changes (data in Table S3). By ranking the selected genes for each group, we verified that the effects of both OP pesticides were predominantly towards the downregulation of transcript expression, that CPF caused noticeable higher effects on gene transcription compared to AZM, and that the effects of CPF became evident earlier, from 6 h on (Fig. 7). The OP AZM was the least effective in changing gene expression at 6 h, approximately ten times lower for downregulation and one-third for upregulation compared to the effects at 24 h or with respect to CPF. We could also verify in the edgeR analysis that the effects of both OP pesticides on the expression of the hypothesis-selected genes positioned them far from the top ranking. None of the pathway-selected genes was within the top-ten, and only three genes were in the 10th–50th or -100th rank for most of the treatments (*gpx1*; *cyp2c19*; and *gstm3*; Table S3), while most of the selected transcripts were within the 500th and 1500th ranks or in the NS group (Fig. 7). These results were in fact similar to those obtained from the non-parametric analysis on the filtered and verified list of transcripts resumed in Fig. 6. Please note that the analysis of top ranked DEGs is not the objective of the present work and is in preparation to be published elsewhere.

### Application of transcriptome data for primer design and PCR analyses

We finally developed PCR assays, with the double purpose of checking the fitness of the fragment sequences determined for the hypothesis-selected genes to design adequate primers, and verifying their response to OP pesticides in ad hoc exposures. We selected 6 genes from the polyamine pathway, 4 genes from the antioxidant and detoxifying pathways, 4 genes from signaling and transcription factors, and 2 HK genes, proceeding to design pairs of primers to develop RT-PCR and qPCR analyses. We succeeded in amplifying 13 of those genes by RT-PCR and could further sequence their products and verify the identities of 8 of them by alignment with the original sequences (Supplementary Table S4). Next, we designed primers suitable for qPCR studies for 10 genes and were able to amplify them all. Thus, we conclude that the de novo assembly of the transcriptome in *R.* *arenarum* was of very good quality and that the annotated gene sequences were adequate for primer design. The general workflow for RT-PCR and qPCR analyses, a detailed description of the methodology, and the corresponding results are presented in the accompanying Supplementary file PCR Methods in Brief.

**Figure 8:**
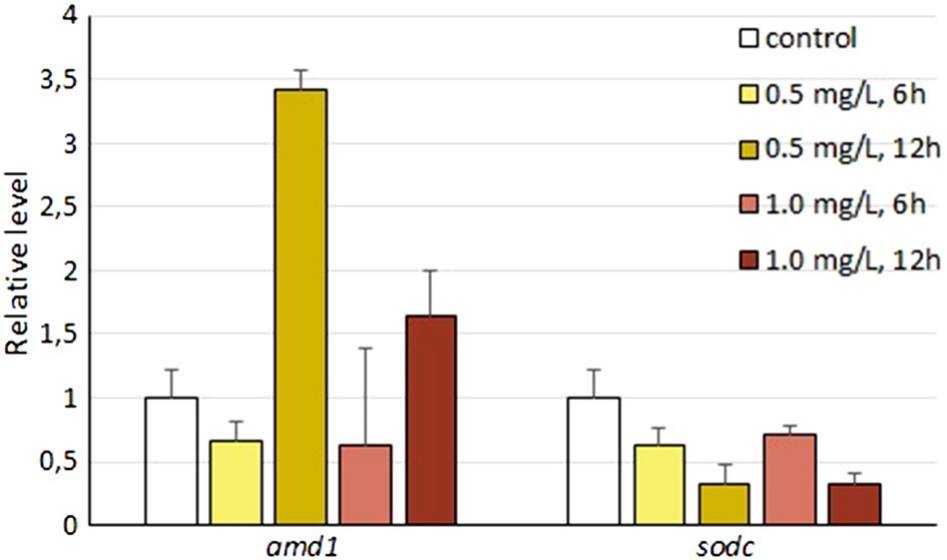
Analysis of gene expression by qPCR in *R. arenarum* larvae exposed to chlorpyrifos. Larvae were exposed to the OP at 0.5 or 1.0 mg/L during 6–12 h. The expressions of two of the selected transcripts were normalized using *actb* and *rl8* as HK genes, applying a geometric mean-methodology, and the relative expression levels were calculated.

Regarding the qPCR results, we found very low levels (high Ct values) for most of the polyamine pathway genes and the *fos* gene, and could not obtain good calibration parameters. We then performed a differential expression analysis of two genes, *amd1* and *sodc*, using *actb* and *rl8* as HK genes in *R. arenarum* larvae exposed to 0.5 and 1.0 mg/L chlorpyrifos for 6–12 h. We applied a geometric means approach to normalize the data with respect to the HK levels and to calculate the relative expression levels. The increase of approximately 3X in *amd1* expression was in very good agreement with a similar increase with both OP pesticides in the transcriptome study (Fig. 8). The decrease in *sodc* expression in the qPCR assay was also coincident with the results of chlorpyrifos-exposed larvae analyzed by RNAseq and transcriptome assembly. Thus, we also conclude that the results of DEG analysis performed on curated and filtered transcriptome data in the hypothesis-driven approach are adequate and reliable.

## Discussion

We succeeded in applying a hypothesis-driven transcript analysis from a transcriptome database of *R. arenarum* larvae exposed to two OP pesticides, with two purposes in mind: (1) to test HK genes currently used in model species and (2) to compare the effects of AZM and CPF on transcript levels of pathways previously described as affected at biochemical or protein expression levels, including an analysis on signaling pathways involved in response regulation. These goals in a native, nonmodel species such as the amphibian *R. arenarum* are a very good example of what transcriptomics analysis can do, solve, or imply for the advance in molecular toxicology when other tools are not readily available^24,30,31^. Considering the results obtained, it is evident that the transcriptomic expression data allow for a screening analysis into selected pathways to decide for further qPCR studies if necessary. Furthermore, we can confirm that transcriptomic information is an essential tool when developing molecular biology assays in nonmodel organisms. Another interesting conclusion involves the high advantage of transcriptomic data on OP pesticides effects as a tool for massive search of potential biomarkers (“biomarkomics”) in environmental risk analyses, which let us propose a set of “best candidates” within the selected pathways in this case.

Our approach to HK genes selection was to look up on previously tested and recommended candidates, in particular on the amphibian model *Xenopus laevis*. Another approach taking advance of the whole transcriptome data would be to select the most stable genes in terms of constant expression, i.e., those showing the least fold-change values. Even if it were a good choice for our particular conditions (exposures to AZM or CPF, up to 24 h, first larvae stages in *R.* *arenarum*) we cannot guarantee that HK genes selected in this way could be used in a generalized way for other situations, even for other OP pesticides. In a comprehensive study conducted with *X. laevis*, *rl8* and *g3pdh* were stable as reference genes during the first embryonic stages, *odc1* was stable in the initial and final stages, and *h4* was adequate throughout embryonic development^27^. In another study in *X. laevis*, e-*ef1a1* and *sub1* were identified as the genes whose expression remained more stable, which would allow their use as reference genes^28^. From a list of 9 genes fulfilling the requirements as HK genes in *R. arenarum*, we successfully tried *actb* and *rl8* in qPCR assays, suggesting that these are good candidates for future qPCR analyses in different environmental studies involving this native species. Thus, we confirm the potential of RNA-Seq data to identify and/or corroborate suitable HK genes, as suggested by other authors^32–34^.

One of the novel and surprising findings in our analysis of the transcriptomic data from *R. arenarum* larvae exposed to sublethal concentrations of AZM and CPF, was that most of the genes from the metabolic and regulatory pathways selected as recognized targets of OP pesticides did not show, in general, remarkable changes in their expression (Fig. 6). This is very interesting, considering that target esterase genes, detoxifying genes, and oxidative stress response genes might *a priori* be expected to be upregulated, as their protein products are either inactivated by OP pesticides or their activities are engaged in xenobiotic transformation and detoxification^13,35–37^. Most of the transcripts showing changes in their levels after OP pesticide exposure did so in a moderate way, and only a few showed fold-changes higher than 2X. Thus, moderate changes in mRNA expression in these genes would be enough for ample response changes in their product activities. Another relevant conclusion extracted from Fig. 6 is that AZM and CPF provoke differential effects of gene transcription, both in quality and in the timing and intensity. Although both OP pesticides have comparable pesticidal potencies in terms of their lethality, their differential impact on gene transcription raises concerns about faster and deeper sublethal damages emerging from undesirable environmental exposure of non-target organisms to CPF. A third observation from Fig. 6 is that the predominant effect of OP pesticides on gene expression is downregulation. This may be a consequence of direct OP effects on signaling and regulatory pathways as we discuss below.

The moderate effects of both OP pesticides on transcription of hypothesis-selected genes are comparatively confirmed with the whole-transcriptome analysis of differential gene expression using edgeR pipeline analysis. The fact that a few of the hypothesis-selected genes show differential expressions that might be useful as biomarker tools, and that they rank among many other DEG not recognized previously as targets of OP pesticides, opens the possibility of searching for novel molecular biomarkers. We are currently engaged in this task, first considering candidates based on the observed fold-changes and the stability and repeatability of the responses and, most important, if these changes may be considered specific for a toxicant or a general pathway response to a stressful situation. With respect to the DEG from the hypothesis-driven approach presented in this work, the best candidates as molecular biomarkers at the transcriptional level would be *gpx1* and *gstm3*, complementary to previous works discussing the enzymatic activities of GPX and GST as biochemical biomarkers for OP pesticides^5,7,13,35^. Other candidates as molecular biomarkers, discussed in more detail below, are *aoc1* and *2*, *ces5a* and *cyp1a1* and *2c19*, as suggested by Fig. 6.

The polyamine metabolism pathway was probably the most affected group of those analyzed in this work, as shown in Fig. 6. The general pattern of the effects of both OP pesticides on polyamine synthesis suggests downregulation through the successive genes involved. Ornithine decarboxylase, S-adenosyl methionine decarboxylase precursor and spermidine-spermine synthase expression were mainly inhibited, except for one *amd1* transcript that showed an induction. These effects are in accord with a reduction in polyamine synthesis or content, as reported in our previous studies. A decrease in putrescine and spermine was observed in *R. arenarum* embryos of the complete operculum stage exposed to AZM^20^. Similarly, CPF downregulated ODC activity and decreased putrescine and spermidine levels in early *R. arenarum* embryos, correlated with the percentage of embryonic developmental arrest^10^. The opposite effect was reported in *R. arenarum* embryos exposed to AZM, where ODC activity and putrescine content were increased^19^. Antizyme transcript expression is also inhibited, possibly as a feedback effect due to ODC inhibition itself. Complementary to these effects on polyamine synthesis, degradation genes such as *aoc1* and *2*, *smox* and *ssat* tend to be downregulated by OP pesticides, probably as a cellular response intended to avoid a lethal drop in polyamine levels^19,38–40^. Nevertheless, the *paox* gene, related to spermidine and spermine degradation, shows an increased expression in larvae exposed to both OP pesticides. This reinforces the reduction of polyamine content along with a reduced expression of synthesizing enzymes. We also found concordance between the transcript expression levels and some of the polyamine-degrading enzymes and previously reported activity values in *R. arenarum* embryos exposed to AZM or CPF, particularly the inhibition of DAO and SMOX activities and the increase in PAOX activity^20^. Therefore, there is a good correspondence between the expression levels found at the transcriptomic level for the regulatory enzymes, some of the polyamine-metabolizing enzyme activities, and the polyamine content in *R. arenarum*. These findings are related to the fine regulation of polyamine metabolism, both at the transcriptional level and at the enzymatic level. On other point of view, the transcript expression levels of these polyamine-related enzymes, such as the different oxidases, would be good candidates for biomarker studies, as proposed for their activities^20^.

Oxidative stress has been reported in different organisms exposed to OP pesticides. In particular, the effects of AZM and CPF on oxidative stress and antioxidant responses at the metabolite and enzymatic levels have been studied in *R. arenarum* development. In fact, we have proposed that there is a link between the effects of OP pesticides on polyamine metabolism and levels, oxidative stress, and the teratogenic effects in *R. arenarum* embryos and larvae^5,7,8,10,14,15,17,19,20,35^. Smirnova et al.^41^ analyzed oxidative stress as the cause of alterations in polyamine metabolism due to the dysregulation of ODC and SSAT; human hepatoma cells chemically induced to increase ROS production showed overexpression of *odc* and *ssat*, which are transcriptionally regulated by Nrf2 through a specific recognition site. The downregulation of *nfe2* mRNA expression with elevated Nrf2 protein levels has been reported in liver pathologies^42^. With respect to the effects of OP pesticides in *R. arenarum* larvae, the downregulation of *nfe2* mRNA expression and the accompanying downregulation at the transcriptional level of *odc*, *ssat* and several antioxidant-detoxifying enzyme genes containing ARE sequences (such as *gsta* and *gstp*) suggest that Nrf2 protein or activity could also be downregulated.

Ma et al.^43^ refer to the alterations in the transcription levels of detoxifying and oxidative stress-related enzymes in the GST and CYP groups in a transcriptomic analysis performed on *Rana chensinensis* exposed to trichlorfon. This OP pesticide upregulated *cyp2c* transcripts but downregulated *cyp3a* and *gstk1*. We report an increase in *gstk1* expression because of CPF exposure in *R. arenarum,* as well as for other *gst* isoforms, such as mu and the microsomal isoforms. This upregulation in several *gst* transcripts is in agreement with the reported increase in GST activity using CDNB as a substrate in *R. arenarum* embryos and larvae after exposure to both AZM and CPF, among other OP pesticides^5,11,17^. The effect on GST activity was associated with an increase in GST-Pi1 protein in *R. arenarum* larvae exposed to arsenic^23^. Other genes under Nrf2 regulation are those belonging to antioxidant defenses, such as *sod*, glutathione peroxidases (*gpx2*, *3*, *6* and *8*), and glutathione reductases (*gsr1*)^43,44^. Accordingly, we observed downregulated levels of *sodc* and *gpx8* accompanied by *nfe2* downregulation. Glutathione synthase *gss* and *cat* also appeared to be downregulated along with *nfe2*, while *sode*, *gpx1*, *3* and *4* were induced, mainly by CPF. In our experience, the antioxidant enzymatic activity response varies greatly in *R. arenarum* embryos and larvae exposed to OP pesticides, showing cycles of induction followed by inhibition attributed to catalytic site inactivation due to ROS attack^8,11,17,21,35^. These cycles in protein contents and enzyme activity may be in turn triggering induction cycles at the transcription levels to restore the required antioxidant protecting levels.

**Figure 9:**
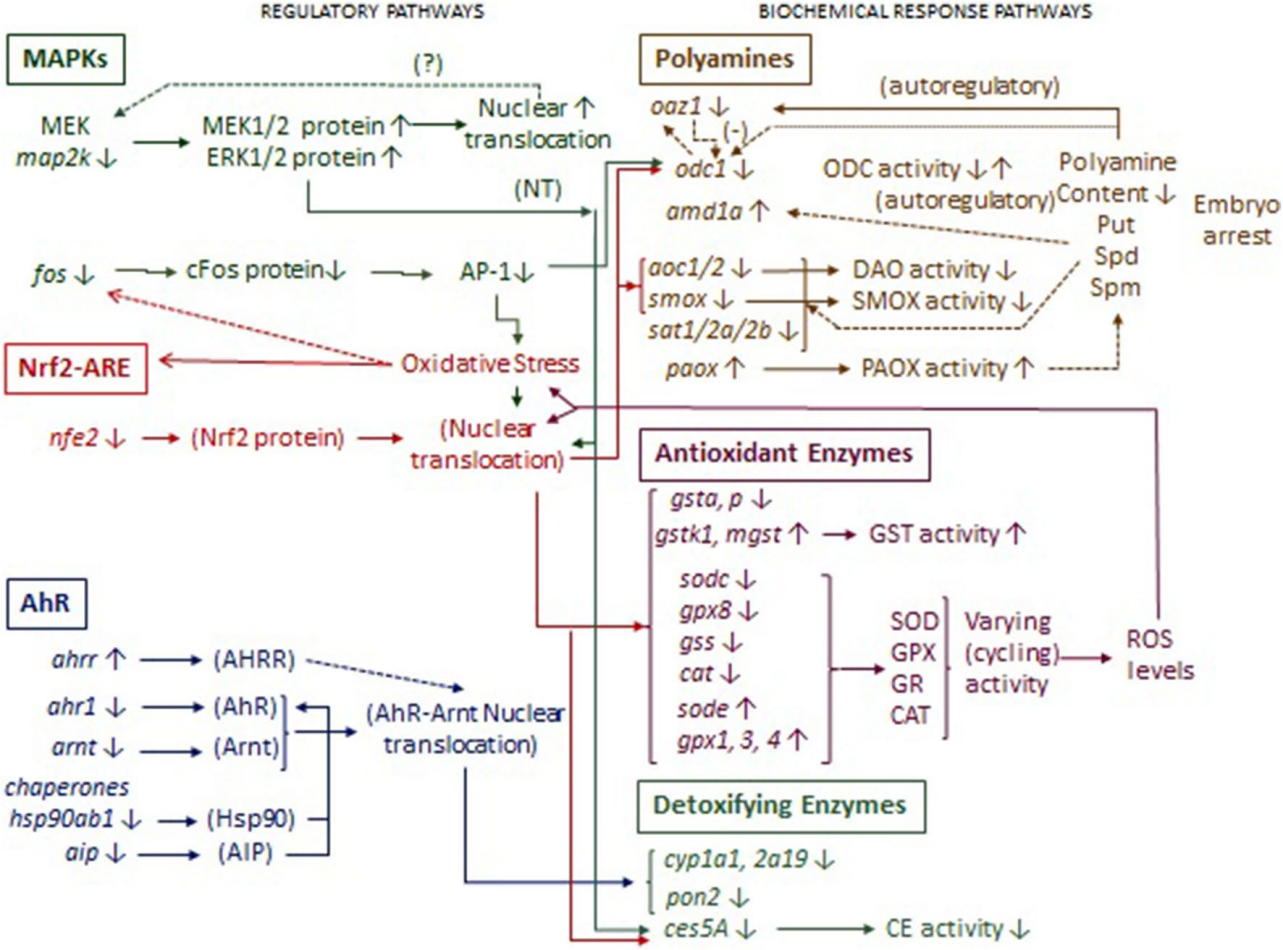
Summary of transcriptomic, biochemical and metabolic findings, and the regulatory crosstalk on the recognized OP-targeted pathways in *R.* *arenarum* larvae. The OP pesticides AZM and CPF interfere in differential ways with the expected responses of protective enzymes, downregulating gene expressions in the regulatory pathways and their active states. The downregulation of MAPKs negatively affects the Nrf2-mediated antioxidant response and a cascade of antioxidant enzyme genes under ARE regulation. Nrf2-ARE and AP-1 downregulation in turn inhibit downstream the synthesis of polyamines essential for normal development and growth. The AhR pathway is negatively regulated by OP pesticides, affecting downstream gene expression of several detoxifying enzymes. In turn, the activities of some of the antioxidant enzymes may act by diminishing ROS impact and oxidative stress caused by OP metabolization, collaborating in the downregulation of Nrf2 pathway. Polyamines also downregulate the transcription and activation of their synthesizing enzymes and upregulate some of their metabolizing enzymes, acting in concert with some transcription factors, and this delicate balance in their levels is affected by AZM and CPF.

We also report here the downregulation of *cyp1a1* and *cyp2a19* for both AZM and CPF exposures, probably as a negative regulation after a previous detoxification response. The genes *cyp1a1/2* are among the genes regulated by the AhR pathway by the binding of activated and nuclear-translocated AhR-Arnt heterodimeric transcription factor to DRE/XRE in their regulatory sequences. Studies carried out with *Sparus aurata* exposed to PCBs have shown a different expression pattern between Ahrr and AhR-Arnt, in accordance with multiple mechanisms contributing to the downregulation of AhR^45^. Our findings coincide with these studies, as we observed induced levels of *ahrr*, while the levels of *arnt* were decreased in *R. arenarum* larvae exposed to both CPF and AZM. Furthermore, the decreased levels of *ahr* and *arnt* are consistent with the decreased expression found in *cyp1a* since the entire mechanism is downregulated. A decrease in *hsp90* and *aip* transcripts, supposing reduced chaperone levels, would also contribute to lower AhR protein levels^46^. The AhR pathway is also involved in the regulation of paraoxonase-1 expression, according to results on exposure to polyphenols and specific inducing ligands^47^. In turn, the *pon2* gene has at least a CRE regulatory sequence for AP-1 regulation linked to the oxidative response and Jnk activation and a PAPR-regulated site related to polyphenol activation and the downregulation of MEK pathway by phosphorylation^48,49^. Our results agree with downregulated MEK (*map2k*), AP-1 (*fos*) and AHR pathways causing *pon2* transcript repression in *R. arenarum* larvae exposed mainly to CPF. Similarly, the xenobiotic/endobiotic detoxifying carboxylesterase family (CES) is transcriptionally regulated by a series of nuclear factors and pathways: AhR, constitutive androstane receptor (CAR), pregnane X receptor (PXR), and Nrf2 are involved mostly in the upregulation of some of these families^50^. Although we were unable to annotate putative transcripts corresponding to CAR and PXR pathways in our *R. arenarum* transcriptome, the downregulation trends followed by AhR and Nrf2 pathways in larvae exposed to AZM and CPF are in line with *ces5a* transcript downregulation. This may be a surprising response to OP pesticides, in the sense that carboxylesterases are recognized suicide “buffer” enzymes that irreversibly react with OP pesticides to protect the primary target acetylcholinesterase in the nervous system, decreasing their activities. This is, in fact, corroborated in toad embryos and larvae exposed to different OP pesticides, including AZM and CPF, in most of our reports in *R. arenarum*^5,8,12,17,51^. Thus, an induction of carboxylesterase and cholinesterase mRNA and/or protein synthesis would be expected to reestablish normal activity levels. Finally, remarking on the complexity of the responses and crosstalk between pathways, we have reported for different developmental states in *R. arenarum* and cell cultures exposed to CPF, other OP pesticides, or arsenic, an increase in Mek1/2 and Erk1/2 proteins, their translocation to the nucleus, and Erk phosphorylation, considering that the MAP kinase pathway regulates the Nrf2-mediated response. The enhanced translocation of Mek to the nucleus due to CPF exposure might be a stress response downregulating the pathway. Furthermore, cFos and cJun proteins are increased after oxidative stress in *R. arenarum* embryos and larvae^12,47,52^. The complex nature of the regulatory crosstalk leading to the prevailing downregulation of biochemical responses in *R. arenarum* larvae exposed to OP, is depicted in Fig. 9. The MAPK pathway is acting upstream the ARE pathway that depends on Nrf2 activation, commanding antioxidant responses; cFos/AP-1 pathway would be partially controlled by oxidative stress but cFos also regulates positively Nrf2 activation. The other main regulatory pathway downregulated by OP exposure is the AhR, which would be acting together with MAPK-Nrf2 on the detoxifying pathways. The three regulatory pathways show a downregulated pattern in response to AZM and CPF, and some of the regulated transcripts such as *mek map2k*, *nfe2*, *fos*, *arnt* and *hsp90ab1* may be good candidates for ‘early-responding’ molecular biomarkers in environmental risk evaluations through conventional qPCR or biomarkomic approaches.

## Concluding remarks

In conclusion, we acknowledge the power of a hypothesis-driven transcriptomic data analysis in selected pathways. First, we identified an appropriate battery of potential housekeeping genes in *R. arenarum* larvae to further analyze gene expression by conventional or quantitative PCR after exposure to pesticides. Second, we were able to visualize the effects of two OP pesticides in *a priori* selected metabolic and signaling pathways and compare them with previous analyses of metabolite and enzyme activity. In our analysis, we infer a gradient of effects since CPF is more potent than AZM and acts earlier on gene transcription. We found mostly downregulation responses in signaling cascades and transcription factors that act upstream the polyamine metabolism pathway and antioxidant responses, which partially coincides with previously well-characterized responses at the protein and/or activity and metabolite levels. Finally, we were able to deepen the insight on the complex crosstalk of the pathways AhR, MAPK and Nrf2 in the antioxidant response, as well as over detoxifying pathways and the polyamine pathway. From the analysis, several gene transcripts emerge as good candidates for biomarker assessments.

## Materials and methods

### Chemicals

High-purity certified standards of azinphos-methyl (98.3% AZM) and chlorpyrifos (CPF; 99.5% purity) were purchased from Chem Service Inc. (West Chester, PA, USA). Standard solutions of 18 g L^−1^ AZM and 1 g L^−1^ CPF were prepared by dissolving the pesticide standards in acetone. The exact concentrations of AZM and CPF in the standard solution were checked by capillary gas chromatography coupled to a nitrogen-phosphorus detector (GC-NPD).

### Biological material

The amphibian *Rhinella arenarum* (Hensel 1867) is widely distributed throughout Argentina^53^. Its life cycle includes two fundamental stages before reaching the adult stage: the embryonic and larval stages. The physiological reproduction of the species in the Upper Valley of Rio Negro and Neuquén (North Patagonia) occurs once a year during the spring months in backwaters and irrigation channels. The reproductive season coincides with the period of greatest pesticide application to protect fruit production. Adult females and males of *R. arenarum* were collected in reference areas, free of pesticide application, in agreement with the corresponding collection permission 040/2020 from the Environment Secretary of Río Negro Province, Argentina. Animals used in this study were maintained and treated with regard to the alleviation of suffering according to recommendations of the Guide for the Care and Use of Laboratory Animals (National Research Council 2011)^54^. The animals were kept in captivity outdoors for 24–48 h until their use. Female ovulation was induced by intraperitoneal injection of 2500 international units (IU) of human chorionic gonadotropin (ELEA Laboratory, Buenos Aires, Argentina.) and embryos were obtained by in vitro fertilization^15^. Embryos were maintained until they reached the complete operculum (CO) stage (stage 25, according to ^55^). Ten days after reaching the CO stage, the larvae were used for acute toxicity assays.

### Acute toxicity assays

The whole protocol was approved by the Faculty Committee for Care and Use of Experimental Animals (CICUAL- Facultad de Ciencias Agrarias Universidad Nacional del Comahue 01/13-7-2020). Larvae were randomly collected to perform the assays. Sublethal concentrations of AZM (0.5 mg L^−1^, 1/20 96 h-LC50; ^6^) and CPF (0.1 mg L^−1^), 1/15 96 h-LC50; ^8^) were selected to carry out exposures for up to 24 h in glass dishes, maintaining a ratio of 1 larva/10 mL in amphibian Ringer's solution with 0.3% acetone (final v/v). These concentrations and higher ones in the order of 1 mg L^−1^ might be transiently found at the irrigation channels in fruit-producing orchards where this species reproduces^4,5^. The exact pesticide concentrations were checked by gas chromatography and nitrogen-phosphorus detection. Control acetone treatment was included to discard possible solvent effects. Treatments were carried out in duplicate, and *R. arenarum* larvae were grown in 10 different glass receptacles to perform AZM/CPF 6 h exposures, AZM/CPF 24 h exposures, and control treatments. From each receptacle, fifteen random larvae were collected and pooled at the corresponding times. Larvae were washed three times with cold Ringer’s solution, placed in 1.5 mL tubes with RNALater^®^ (Thermo Fisher Scientific Inc.) and stored at − 20 °C until processed.

### RNA extraction, cDNA library generation and massive parallel sequencing

RNA extraction, cDNA library generation and massive parallel sequencing were carried out as described by Ceschin et al.^25^. Briefly, total RNA of each sample was extracted, and the cDNA library for transcriptome analysis was prepared. The ten library samples were normalized to 10 nM cDNA to be sequenced on a HiSeq 1500 Illumina platform, generating nonstrand specific “paired-ends” (PE) 2 × 100 bp readings.

### Bioinformatic construction of *R. arenarum* transcriptome and statistical analysis of expression levels

A detailed description of the de novo transcriptome assembly, annotation and gene prediction was previously provided by Ceschin et al.^25^. Briefly, the readings obtained by massive sequencing of the *R. arenarum* transcriptome were aligned with Bowtie2 v2.3.5^56^ against TSA: GHCG00000000.1 (BioProject PRJNA485066), and transcript expression quantification was performed using RSEM v1.3.0^57,58^. Expression values were normalized by the TMM method using the R and edgeR packages^59,60^. The threshold values currently applied for the selection of DEGs were a fold-change of 2 or one-half, respectively for induction or downregulation, with a *p*-value ≤ 0.05. Once the appropriate HK transcripts were identified, the TMM values were standardized both by the average of the selected reference genes and by the respective control values. Finally, a nonparametric analysis was performed by the median and Kruskal-Wallis tests to assess significant differences or tendencies using the exact p-values. Raw data, standardization steps and statistical analyses are available in Supplementary Data File II.

The validation of transcript expression results was performed by qPCR analysis on *R. arenarum* larvae exposed to CPF, following the same procedures described above for the treatments and extraction of RNA. The different steps for PCR development are detailed in the “Supplementary file PCR data methods in brief”.

### ARRIVE guideline statement

This study is reported in accordance with ARRIVE guidelines (https://arriveguidelines.org).

## Supplementary Information

Supplementary Information 1.

Supplementary Information 2.

Supplementary Information 3.

Supplementary Information 4.

## Data Availability

All data generated and analyzed during this study are included in the Supplementary Data Files I and II.
