# Supplementary material for: Hypothesis-driven dragging of transcriptomic data to analyze proven targeted pathways in Rhinella arenarum larvae exposed to organophosphorus pesticides
Source: Sci Rep. 2022 Oct 21;12:17712. doi: 10.1038/s41598-022-21748-6 (PMC9587056; doi:10.1038/s41598-022-21748-6)
Supplement: Supplementary file 3 — Supplementary Information 3. [file 41598_2022_21748_MOESM3_ESM.pdf]

Supplementary Tables

Natalia S. Pires, Cecilia I. Lascano, Julia Ousset, Danilo G. Ceschin and Andrés Venturino

Hypothesis-driven dragging of transcriptomic data to analyze proven targeted pathways in *Rhinella arenarum* larvae exposed to organophosphorus pesticides.

Table S1. List of available annotated genes from R. arenarum transcriptome

GROUP A, HOUSEKEEPING GENES	
Elongation Factor 1-alpha	*ef1a0, 1a1, 1a2*
Elongation Factor 1-beta	*ef1b*
Elongation Factor 1-gamma A	*ef1g-a*
Elongation Factor 1-delta	*ef1d*
Glyceraldehyde-3-phosphate dehydrogenase	*g3pdh*
60S ribosomal protein L8	*rl8*
Tubulin alpha chain	*tuba, tuba1*
Tubulin beta chain	*tubb, tubb4b*
cytoplasmic Actin	*actb*
Actin alpha sarcomeric	*acta4*
GROUP B, POLYAMINE METABOLISM GENES	
Ornithine decarboxylase	*odc1*
Ornithine decarboxylase antizyme	*oaz1, 2*
Antizyme inhibitor	*azin1, 2*
S-adenosyl methionine decarboxylase precursor	*amd1*
Spermidine synthase	*srm*
Amiloride-sensitive Cu-containing amine oxidase / diamine oxidase	*aoc1* to *4*
N(1)-acetyl-spermine/spermidine oxidase	*paox*
Spermine oxidase	*smox*
Spermidine/spermine N1 acetyltransferase	*ssat*
GROUP C, OXIDATIVE STRESS - ANTIOXIDANT RESPONSE GENES	
Superoxide dismutases	*sod c, d, e, 5*
Catalases	*cat*
Glutathione peroxidases	*gpx1* to *8*
Glutathione reductase	*gsr*
Glutathione synthetase	*gss*
GROUP D, OP- METABOLIZING AND TARGET ENZYME GENES	
Acetyl cholinesterases	*ache*
Butyryl cholinesterases	*bche*
Carboxylesterases	*ces3b, ces5a*

Serum paraoxonase/arylesterase	*pon2*
Glutathione transferases alpha	*gsta*
Glutathione transferases kappa	*gstk*
Glutathione transferases mu	*gstm*
Glutathione transferases omega	*gsto*
Glutathione transferases pi	*gstp*
Glutathione transferases theta	*gstt*
Glutathione transferases microsomal forms	*mgst1* to *3*
Cytochrome P450 1A1	*cyp1a1*
Cytochrome P450 2C19	*cyp2c19*
GROUP E, TRANSCRIPTION FACTORS AND PHOSPHORYLATION CASCADES	
Mitogen-Activated protein kinase pathway	
MAPKK/ MEK	*map2k1, k2*
MAPK	*mapk14* (p38), *mapk8/jnk*
Aryl hydrocarbon receptor pathway	
Aryl hydrocarbon receptor	*ahr*
AhR repressor	*ahrr*
nuclear translocator	*arnt*
heat-shock protein 90	*hsp90ab1*
Aryl-hydrocarbon-interacting proteins	*aip* and *aipl1*
Prostaglandin E synthase 3	*ptges3*
Transcription factor AP-1 JUN	*jun 1, 2, b, d*
Proto-oncogene c-Fos	*fos*
Nuclear factor erythroid 2-related factor 2	*nfe2*

Table S2. Gene transcripts showing no significant or not relevant changes in *R. arenarum* larvae after exposure to OP pesticides.

Transcript			Fold-change			p-value
PA pathway						
	control	AZM6	AZM24	CPF6	CPF24	
oaz2	1 ± 0.04	1.09 ± 0.00	1.04 ± 0.05	1.04 ± 0.04	1.09 ± 0.03	0.35
azin1	1 ± 0.07	1.08 ± 0.10	0.85 ± 0.03*	1.11 ± 0.09	0.95 ± 0.05	0.04*
azin2	1 ± 0.11	0.90 ± 0.12	0.77 ± 0.12	0.88 ± 0.29	0.75 ± 0.08	0.41
aoc2	1 ± 0.06	0.52 ± 0.15	0.54 ± 0.02	0.31 ± 0.10	0.46 ± 0.05	0.17
aoc3	1 ± 0.02	0.68 ± 0.33	1.08 ± 0.14	1.13 ± 0.40	1.70 ± 0.01	0.29
aoc4	1 ± 0.37	1.19 ± 0.03	0.98 ± 0.16	1.48 ± 0.07	1.04 ± 0.37	0.40
paox2	1 ± 0.08	0.89 ± 0.07	1.00 ± 0.05	1.04 ± 0.10	0.91 ± 0.03	0.51
Antioxidant enzymes						
gpx2	1 ± 0.26	1.02 ± 0.10	0.46 ± 0.02	0.70 ± 0.09	0.84 ± 0.40	0.41
gpx7	1 ± 0.39	1.00 ± 0.27	0.66 ± 0.10	0.70 ± 0.12	0.86 ± 0.08	0.41
gsr	1 ± 0.12	0.75 ± 0.04	0.74 ± 0.02	0.73 ± 0.02	0.69 ± 0.08	0.41
Detoxifying enzymes						
ces3b	1 ± 0.08	0.65 ± 0.09	0.84 ± 0.05	0.64 ± 0.17	0.87 ± 0.26	0.35
gstc	1 ± 0.06	0.92 ± 0.03	0.94 ± 0.00	0.98 ± 0.05	1.05 ± 0.05	0.32
gstm1	1 ± 0.07	1.00 ± 0.10	0.81 ± 0.10	0.73 ± 0.10	0.90 ± 0.12	0.24
gstp1-2	1 ± 0.00	1.07 ± 0.06	1.15 ± 0.03	1.08 ± 0.05	1.15 ± 0.05	0.20
Signaling pathways and transcription factors						
map2k1	1 ± 0.02	1.03 ± 0.01	0.95 ± 0.02	0.92 ± 0.02*	0.97 ± 0.01	0.09*
map2k2-2	1 ± 0.04	0.91 ± 0.05	0.81 ± 0.05	0.91 ± 0.03	0.87 ± 0.03	0.26
mapk14	1 ± 0.01	0.92 ± 0.04	0.87 ± 0.03*	0.89 ± 0.03*	0.94 ± 0.00	0.09*
mapk8	1 ± 0.04	1.00 ± 0.01	0.92 ± 0.06	1.02 ± 0.06	0.89 ± 0.01	0.32
jun1	1 ± 0.14	1.13 ± 0.06	1.16 ± 0.01	1.23 ± 0.15	0.83 ± 0.13	0.27
junb	1 ± 0.17	1.17 ± 0.33	1.22 ± 0.24	1.37 ± 0.06	0.80 ± 0.13	0.40
jund1	1 ± 0.05	0.98 ± 0.04	0.81 ± 0.05	0.94 ± 0.08	0.86 ± 0.01	0.94
ahr	1 ± 0.06	0.85 ± 0.04	0.90 ± 0.26	0.78 ± 0.15	0.74 ± 0.09	0.14
aip	1 ± 0.03	0.98 ± 0.04	1.00 ± 0.00	1.08 ± 0.03	1.00 ± 0.01	0.35
ptges3	1 ± 0.09	0.83 ± 0.05	0.68 ± 0.02	0.87 ± 0.06	0.79 ± 0.03	0.14

AZM: azinphosmethyl; CPF: chlorpyrifos. *Significant, not relevant changes

TABLE S3. Comparison between non-parametric tests and edgeR performances.

Transcript (I)[1]	KW/ Median p value (n)[2]	paired edgeR (Treated vs Control) analysis							
		p values[3]				DEG Ranking[4]			
		AZM6	AZM24	CPF6	CPF24	AZM6	AZM24	CPF6	CPF24
PA pathway									
odc1	0.09 (6)	0.33	0.44	1	0.42				
srm	0.09	0.51	0.28	0.11	0.05				#-1281
oaz1	0.05 (4)	0.79	0.001	0.002	0.03		#-819	#-1038	#-1429
amd1a	0.09	Data not found in edgeR							
amd1b	0.09	0.24	0.06	0.06	0.07		#-1206	#-1330	#-1340
aoc1 (4)	0.0001 (8)	0.14	0.11	0.01	0.13			#-246	
		0.13	0.02	0.008	0.12		#-417	#-363	
		0.06	0.01	0.002	0.01	#-62	#-411	#-253	#-422
		0.30	0.07	0.007	0.05		#-577	#-222	#-529
paox	0.09	0.43	0.95	0.26	0.94				
smox	0.09	0.49	0.56	0.40	0.76				
ssat1	0.09	0.18	0.0009	0.002	0.001		#-472	#-843	#-587
ssat2a	0.09	0.12	0.02	0.36	0.0008				
ssat2b	0.08	0.47	0.72	0.73	0.55				
OXIDATIVE STRESS									
gpx1	0.08	0.77	$5x10^{-7}$	$1x10^{-6}$	$2x10^{-5}$		#+24	#+58	#+96
gpx3	0.01 (4)	0.56	0.63	0.07	0.04			#+375	#+210
gpx4	0.07 (4)	1	0.40	0.17	0.14				
gpx8b	0.09	0.47	0.13	0.04	0.12			#-796	
gss	0.09	0.65	0.80	0.64	0.71				
sodc	0.09	0.54	0.05	0.005	0.09		#-1310	#-1071	#-1468
sode (2)	0.0007(12)	0.31	0.54	0.15	0.36				
cat (2)	0.09 (4)	0.64	0.28	0.23	0.17				
DETOXIFYING ENZYMES									
ces5a	0.09	Data not found in edgeR							
pon2	0.05	0.74	0.74	0.20	0.75				

cyp1a1	0.007 (4)	0.90	0.90	0.80	0.60				
cyp2c19	0.09	0.06	0.003	0.06	0.003	#-41	#-65	#-656	#-59
gsta3	0.09	0.01	0.09	0.08	0.59	#-72	#-1331	#-1438	
gstk1	0.04 (4)	0.66	0.58	0.13	0.03				#+285
gstm3	0.09	0.89	0.0001	0.0003	0.0001		#+38	#+89	#+13
gsto1	0.05 (6)	0.41	0.74	0.38	0.25				
gstp1 (2)	0.0001 (8)	0.23	0.003	0.009	0.01		#-346	#-674	#-728
		0.98	0.04	0.05	0.01		#-997	#-1208	#-719
gstp2	0.08	0.31	0.01	0.004	0.24		#-1236	#-1177	
gstt3	0.09	0.95	0.01	0.004	0.04		#-1133	#-1012	#-1404
mgst1	0.09	0.88	0.01	0.02	0.002		#+294	#+430	#+223
mgst2-1	0.09	0.18	0.99	0.37	0.19				
mgst2-2	0.09	0.95	0.28	0.44	0.34				
mgst3-1	0.09	0.88	0.02	0.03	0.003		#+297	#+432	#+215
mgst3-2	0.09	0.93	0.95	0.80	0.15				
TRANSCRIPTION FACTORS, SIGNALING									
map2k2	0.09	0.89	0.38	0.52	0.74				
nfe2	0.09	0.21	0.01	0.01	0.008		#-96	#-1051	#-948
arnt	0.09	0.001	0.35	0.26	0.05				
arnt2	0.09	0.30	0.90	0.0006	0.60				
ahrr	0.09	0.80	1	0.90	0.50				
hsp90ab1	0.09 (4)	0.47	0.91	0.25	0.01				#-1409
aipl1	0.09	0.31	0.80	0.40	0.25				
fos	0.08	0.01	0.25	0.27	0.70				

The p-values for annotated transcripts in hypothesis-driven selected pathways, showing statistically significant differential expressions in non-parametric tests (filtered database), were included for comparison with edgeR analysis performed on not filtered database. AZM: azinphos methyl; CPF: chlorpyrifos; 6 and 24 h of treatment.

[1] (I) isoforms for one annotated gene are indicated by separate in edgeR analysis, if more than one

[2] KW, Kruskal-Wallis ANOVA and Median non-parametric tests, best p value for (n) number of data per treatment, when n is higher than 2.

[3] edgeR p-value ranges when more than one fragment and/or isoform were determined for a single annotated gene, for the different pairs of treatments. Red numbers indicate no coincidence with non-parametric statistics for the same pair of treatments.

4 Ranking only for significant DEG; #- for downregulation; #+ for upregulation. Total DEG in each treatment: AZM6 #- 185, #+ 121; AZM24 #-1379, #+ 374; CPF6 #- 1513, #+ 492; CPF24 #- 1504, #+ 347.

Table S4. Summary of transcripts and steps performed to develop further expression analysis by RT-PCR and qPCR

Transcript	RT-PCR			qPCR		
	Primer	Amplification	Sequence Validated	Primer	Amplification	Expression Testing
Polyamine pathway						
odc1	Y	Y	Y	Y	(Y)	-
paox	Y	Y	Y	Y	(Y)	-
aoc1	Y	Y	Y	Y	(Y)	-
smox	Y	N	-	Y	(Y)	-
amd1	Y	Y	N	Y	Y	Y
srm	Y	Y	Y	Y	(Y)	-
Antioxidant response						
cat	Y	Y	N	-	-	-
gsr	Y	N	-	-	-	-
gstp	Y	Y	N	-	-	-
sodc	Y	Y	N	Y	Y	Y
Signaling and transcription factors						
fos	Y	Y	Y	Y	(Y)	-
nfe2	Y	Y	N	-	-	-
jun	Y	Y	Y	-	-	-
Mapk8/jnk	Y	N	-	-	-	-
Housekeeping genes						
actb	Y	Y	Y	Y	Y	Y
rl8	Y	Y	Y	Y	Y	Y

Y indicates succesful step; (Y) indicates amplification at high cycle numbers that difficulted validation and further testing of differential expression levels. Expression testing refers to transcript expression analysis by qPCR in *R. arenarum* larvae exposed to azinphos methyl and chlorpyrifos.
