# Supplementary material for: Hypothesis-driven dragging of transcriptomic data to analyze proven targeted pathways in Rhinella arenarum larvae exposed to organophosphorus pesticides
Source: Sci Rep. 2022 Oct 21;12:17712. doi: 10.1038/s41598-022-21748-6 (PMC9587056; doi:10.1038/s41598-022-21748-6)
Supplement: Supplementary file 4 — Supplementary Information 4. [file 41598_2022_21748_MOESM4_ESM.pdf]

Supplementary PCR Data Methods in brief

Hypothesis-driven dragging of transcriptomic data to analyze proven targeted pathways in *Rhinella arenarum* larvae exposed to organophosphorus pesticides.

Natalia S. Pires[1], Cecilia I. Lascano[1], Julia Ousset[1], Danilo G. Ceschin[2] and Andrés Venturino[1]

[1] Centro de Investigaciones en Toxicología Ambiental y Agrobiotecnología del Comahue (CITAAC), Universidad Nacional del Comahue-CONICET. Buenos Aires 1400, Neuquén (8300), Argentina
[2] Unidad de Bioinformática Traslacional, Centro de Investigación en Medicina Traslacional Severo Amuchástegui, Instituto Universitario de Ciencias Biomédicas de Córdoba, Av. Naciones Unidas 420, Córdoba (5000), Argentina.

Workflow of PCR molecular studies to validate annotated transcripts and analyse gene expression in *R. arenarum*

Based on the depurated transcript sequences annotated from the *R. arenarum* transcriptomic experiment [1], primers were designed using the tools Primer 3 and PrimerBLAST [2–4]. Considering that there are pathways known to be impacted by exposure to organophosphorus pesticides, primer design was focused on the polyamine metabolism pathway, the antioxidant and detoxifying system, and transcription factors and signaling pathways. The following table shows the primers that were designed to validate the annotated transcripts through RT-PCR and product sequencing.

PRIMER	GENE NAME	PATHWAY	SEQUENCE
ODC1_F<br>ODC1_R	Ornithine decarboxylase	Polyamine Metabolism (PM)	CTGGTAGGTCGAAACGCTCA<br>ACTTTCCAGTTGACTCCGGC
PAOX_F<br>PAOX_R	Acetyl spermidine/spermine oxidase	PM	AGGGCCATCTGCTCTTTCAC<br>CGTGGACGAGGCTTGTAACT
AOC1_F<br>AOC1_R	Diamine oxidase	PM	GAGGGCCTATTGGAGACGTG<br>AATGCAGTGGACGACCTGTT
SMOX_F<br>SMOX_R	Spermine oxidase	PM	TCCTTCCTGGAGAACCCCTTT<br>TGTGGTACGAAAACGCGTCA
AMD1_F<br>AMD1_R	S-adenosylmethionine decarboxylase proenzyme	PM	ATGAGGCCAAGACCGTCAAC<br>CGTTGAACTGAGCGAACTGG
CAT_F<br>CAT_R	Catalase	Antioxidant System (AS)	ACCAGAGAGATGGGTTCAG<br>TGCCAGTTTGTCCAGTTCAG
GSR_F<br>GSR_R	Glutathione reductase	AS	CCCATTTTGATAGCGACGGC<br>GGCAGAGCACTTTTGACACC
SODC_F<br>SODC_R	Superoxide dismutase	AS	GTCCAGTTGGGGCTCTTACC<br>TGTGCTGGTACAGCAGTAAAC
GSTP_F<br>GSTP_R	Glutathione S-transferase Pi	DS	CACCAGGTTGTAGTCGGCAA<br>GGCAGCCCTCATAGACATGG
FOS_F<br>FOS_R	Fos proto-oncogene	Transcription Factor (TF)	AGCATCGCTATGGGTCACAG<br>ACTCCTGGGTTCCGGGATAA
NFE2_F<br>NFE2_R	Nuclear factor erythroid 2-related factor 2	TF	AGAAGGAATTGTACCCGCCG<br>GATGTCCCTGACCAAAGCCA
JUN-F<br>JUN_R	Jun proto-oncogene	TF	CCGCTCGGCTTTTATCCTCT<br>ACCCAGTTCCTCTGCCCTAA
JUNK_F<br>JUNK_R	c-Jun N-terminal kinase	TF	TTGCTGCTGCTCACTCTTTG<br>CGATCTGGAGTTTGTTCCT
ACTB_F<br>ACTB_R	Beta Actin	Housekeeping (HK)	TCCTGACCCTGAAGTACCCC<br>GGTGCCCATCTCCTGCTCGA

With these primers, RT-PCR were performed with R. arenarum samples.The following pictures show representative agarose gel electrophoresis of the selected genes, along with the product size in base pairs (bp):

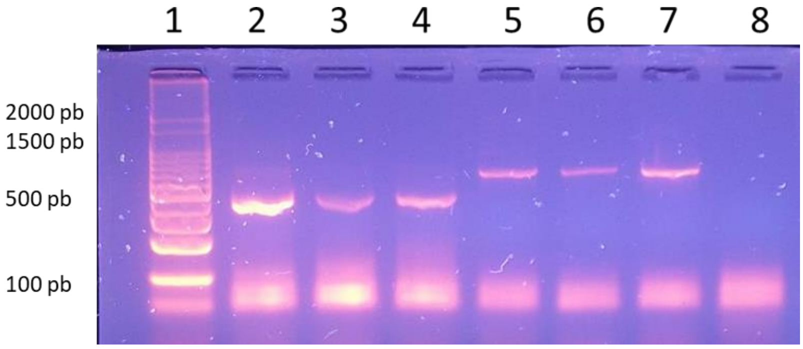

PCR amplification of *actb* and *odc1*. Line 1: molecular weight marker. Lines 2-4: *actb* amplified by RT-PCR from liver of adult *R. arenarum* individuals; 500 bp. Lines 5-7: *odc1* amplified by RT-PCR from liver of adult *R. arenarum* individuals; 800 bp.

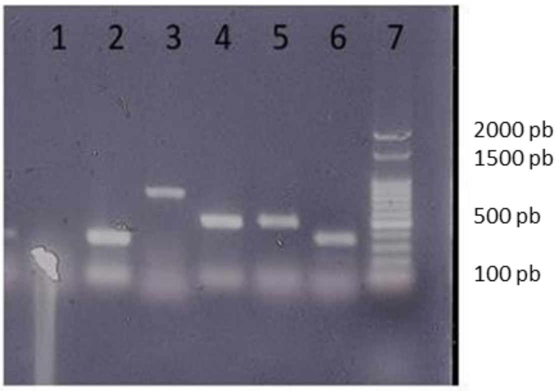

PCR amplification of *jun*, *paox*, *actb* and *cat*. Line 1: negative control. Line 2: *jun*, 200 bp. Line 3: *paox*, 700 bp. Lines 4 and 5: *actb*, 500 bp. Line 6: *cat*, 300 bp. Line 7: molecular weight marker.

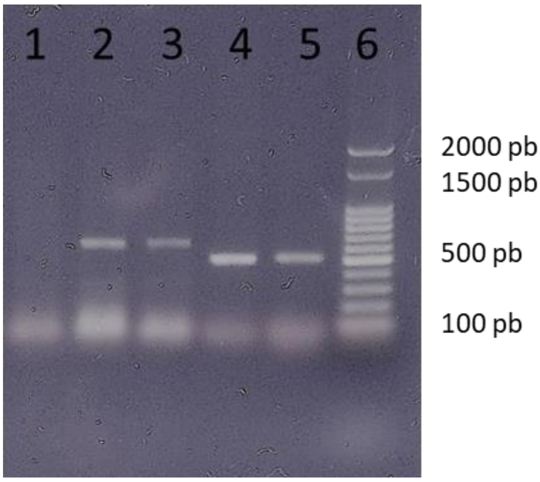

PCR amplification of *nfe2* and *sodc*. Line 1 negative control: Lines 2 and 3: *nfe2*, 600 bp. Lines 4 and 5: *sodc*, 500 bp. Line 6: molecular weight marker.

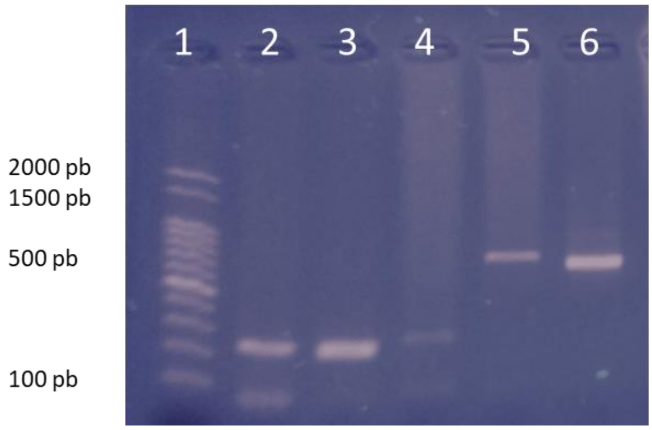

PCR amplification of *jun*, *aoc1* and *gstp*. Line 1: molecular weight marker. Lines 2 and 3: *gstp*, 200 bp. Line 5: *jun*, 700 bp. Line 6: *aoc1*, 700 bp.

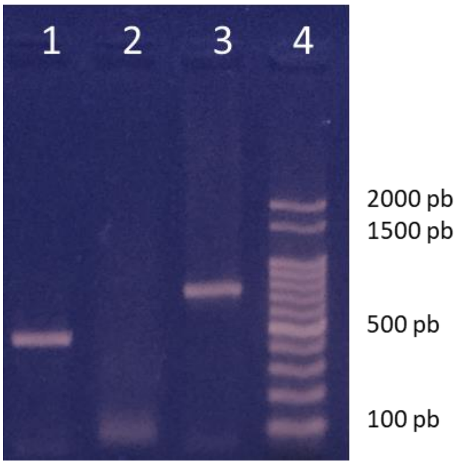

PCR amplification of *amd1* and *jun*. Line 1: *amd1*, 500 bp. Line 2: non amplified sample. Line 3: *jun*, 700 bp. Line 4: molecular weight marker.

The genes that were successfully amplified by RT-PCR were sequenced and aligned using a free version of Geneious Prime. *paox*, *fos*, *odc1*, *aoc1* and *jun* displayed 100% homology with the shown portions of the transcriptome-assembled transcripts (blue numbers in the accompanying figure). No homology was found between the PCR-amplified genes *cat*, *nfe2*, *sodc*, *amd1* and *gstp* and the transcriptome information.

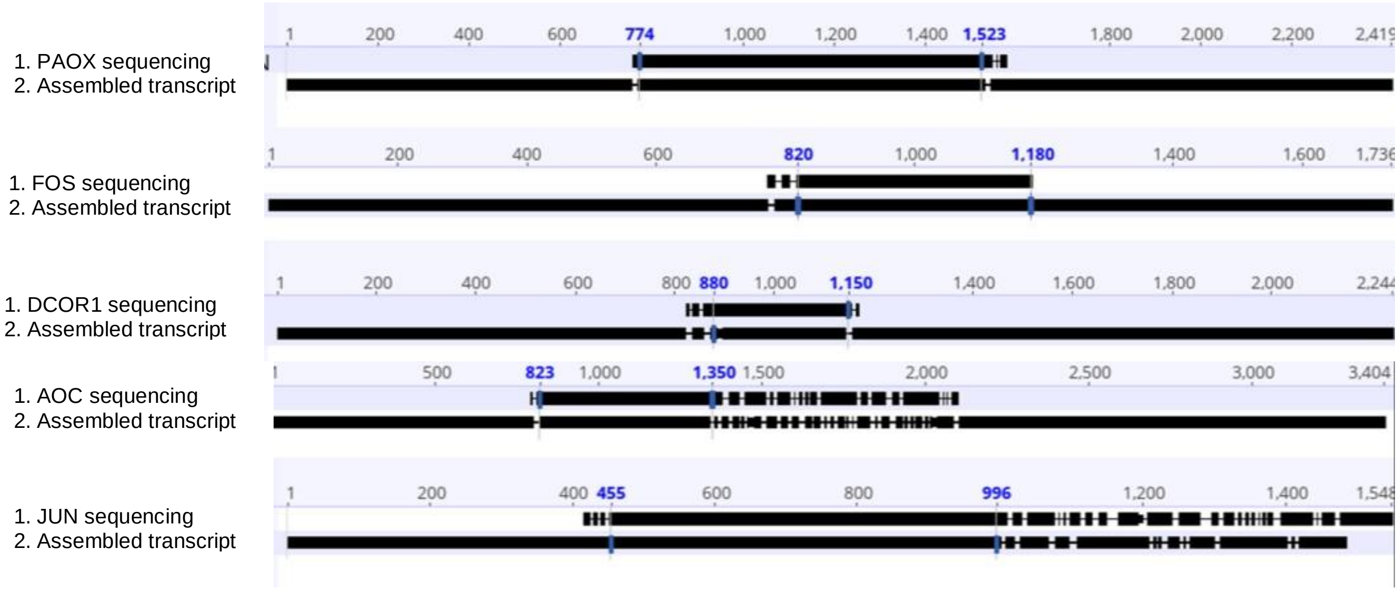

Quantitative PCR analyses applied to selected *R. arenarum* transcripts

Our next task was to design new primers suitable for qPCR studies. The following table shows the designed primers:

PRIMER	GENE NAME	PATHWAY	SEQUENCE
PAOX_F<br>PAOX_R	Acetyl spermidine/spermine oxidase	PM	CTGGAAACTGCTCACCATCTTCAC<br>AAGGGCTACCAAGCTCTACTAGAC
AOC1_F<br>AOC1_R	Diamine oxidase	PM	CTGCTGCTCCATCACTTG<br>CTCTGCCAGGGTCGTAATAG
SMOX_F<br>SMOX_R	Spermine oxidase	PM	TCCTTCCTTCCTGGAGAAC<br>GAGGCTGAAACTGGCTATG
SRM_F<br>SRM_R	Spermidine Synthase	PM	AAGACTCTGTTCCCTGTGGTAG<br>ATGCTGCCCGATGAATGTTG
ODC1_F<br>ODC1_R	Ornithine decarboxylase	PM	TCCGTAGACACCATCGTTGAC<br>TGTTGCTTCAGCCTTCAC
AMD1_F<br>AMD1_R	S-adenosylmethionine decarboxylase proenzyme	PM	ACCAGACCATCAGGAATACC<br>CATGCGTCCCATGCAATAAG
SODC_F<br>SODC_R	Superoxide dismutase	AS	GCAACGCCATCTTTGGAGG<br>GCACTATCAACGGGCTGAC
FOS_F<br>FOS_R	Fos proto-oncogene	TF	ACCACCTACACCACCTCGTTTGTC<br>ACTGCTCGTTGCTGCTGCTTC
ACTB_F<br>ACTB_R	Beta actin	HK	GCTGTGCTGTCCCTGTATG<br>CAAGTCCAGACGCAGGATG
RL8_F<br>RL8_R	Ribosomal protein L8	HK	GTGGCTATGAACCCTGTAGAA<br>ACGACGAGCAGCAATAAGAC

We developed qPCR protocols with these primers to assess transcript expression in *R. arenarum*. Primer validation through standard curves and dynamic range calculation was carried out for the designed primers. Primers to amplify *rl8*, *amd1*, *actb* and *sodc* were validated (see next table). Primers designed to amplify *fos*, *paox*, *odc1* *aoc1*, *smox* and *srm* could not be validated, as their expression levels were very low and amplification began between Ct 28 and Ct 33. Pre-amplification cycles and addition of DMSO were attempted, but no positive results were obtained.

PARAMETER	***actb***	***amd1***	***sodc***
Slope	-2.191	-3.561	-3.173
Y-intercept	31.78	34.58	34.51
Efficiency	1.86	0.91	1.07
$R^{\wedge 2}$	0.986	0.996	0.984

Initial trials were developed to test the applicability of the *R. arenarum* transcriptome database in qPCR assays, using the validated primers and protocols for *sodc* and *amd1* genes using *actb* and *rl8* as HK genes in *R. arenarum* larvae exposed to chlorpyrifos. A pipeline similar in several aspects to the GeNorm algorithm, was used to analyse the expression results: First, geometric mean calculations for both HK were performed, using Ct values for each condition and replicate. After calculating delta Ct values using the calculated geometric mean HK value, we performed the geometric mean calculations on the sample replicates. We obtained an average standard deviation for the genes of interest from the mean deviation of their ranges of variation. We then normalized to control-zero values, as the delta-delta Ct, proceeding then to obtain the relative expression levels in each condition for the genes of interest. The results of this trial are shown in the following figure:

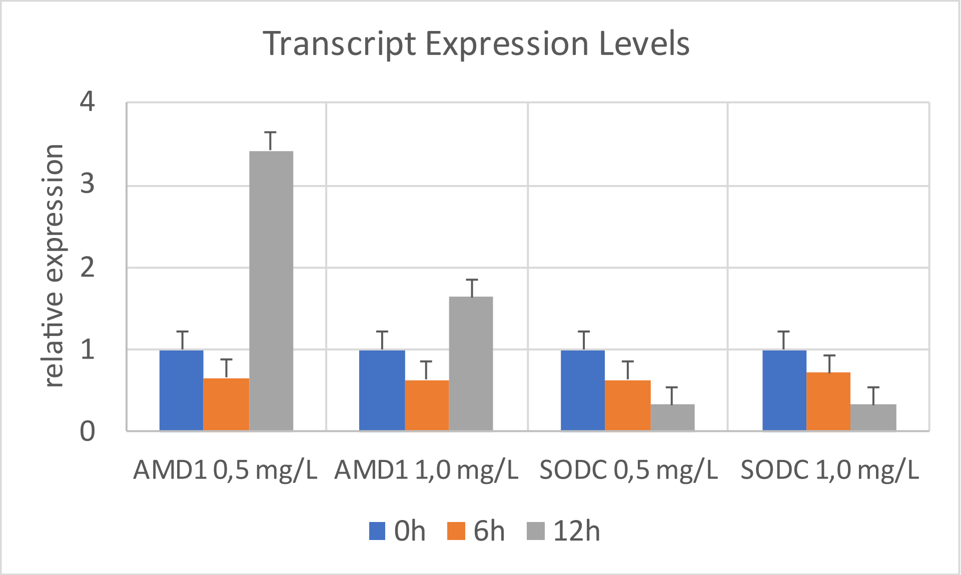

Our preliminary results support the conclussion that the *de novo* assembled *R. arenarum* transcriptome provides adequate information to choose a battery of potential housekeeping genes and further analyze gene expression by quantitative PCR on a series of genes from hypothesis-selected pathways.

References

1. Ceschin, D. G., Pires, N. S., Mardirosian, M. N., Lascano, C. I. & Venturino, A. The Rhinella arenarum transcriptome: de novo assembly, annotation and gene prediction. *Sci. Rep.* **10**, (2020).
2. Untergasser, A. *et al.* Primer3--new capabilities and interfaces. *Nucleic Acids Res.* **40**, e115 (2012).
3. Koressaar, T. & Remm, M. Enhancements and modifications of primer design program Primer3. *Bioinformatics* **23**, 1289-91 (2007).
4. Ye, J. *et al.* Primer-BLAST: A tool to design target-specific primers for polymerase chain reaction. *BMC Bioinformatics* **13**, 134 (2012).
